# Ion-coupled transfersome complexes for enhanced transdermal NAD^+^ repletion and mitigation of cellular senescence signatures

**DOI:** 10.1016/j.mtbio.2026.103218

**Published:** 2026-05-06

**Authors:** Seongsu Kang, Shibo Wei, Bon Il Koo, Yunju Jo, Junhyeon Park, Yingqi Xue, Sanghyun Ye, Jiwon Park, Byung Woo Hwang, Jin Hyun Kim, Euitaek Jeong, Juewon Kim, Nea-Gyu Kang, Seung-Hyun Jun, Dongryeol Ryu

**Affiliations:** a LG Household and Health Care R&D Center, Seoul, Republic of Korea; b Department of Biomedical Science and Engineering, Gwangju Institute of Science and Technology (GIST), Gwangju, Republic of Korea; c Department of Microbiology, Wonkwang University School of Medicine, Iksan, Republic of Korea; d Department of Physiology, Konkuk University College of Medicine, Chungju, Republic of Korea

**Keywords:** NAD^+^, Nanovesicles, Ion-coupled transfersome, Transdermal delivery, Skin aging, Spatially resolved transcriptomics, Human skin explants

## Abstract

Nicotinamide adenine dinucleotide (NAD^+^) is a pivotal coenzyme whose decline drives mitochondrial dysfunction and senescence. However, NAD^+^ delivery to skin is limited by poor stability and tissue accessibility. Here, we develop an ion-coupled NAD^+^ transfersome (ICoN) that stabilizes NAD^+^ and enables efficient, non-invasive transdermal delivery. ICoN restores intracellular NAD^+^ levels and reverses transcriptomic programs associated with DNA damage, oxidative stress, and senescence *in vitro*. Functionally, ICoN promotes mitochondrial functional integrity and sustains proliferative and migratory competence under senescent and NAD^+^-depleted conditions. In *ex vivo* porcine and human skin explants, ICoN achieves superior intratissue NAD^+^ penetration while attenuating senescent signatures compared with free NAD^+^ or precursors. Integrated high-spatial-resolution transcriptomic profiling and bulk transcriptomic analyses of UV-stressed human skin reveal that ICoN mitigates histological disruption and senescence-associated transcriptional programs at the tissue level. Moreover, ICoN extends lifespan and enhances healthspan in *C*. *elegans*. These findings characterize ICoN as a transdermal NAD ^+^ delivery platform supporting localized NAD ^+^ restoration in the context of skin aging.

## Highlights

• Ion-coupled transfersomes stabilize NAD^+^ for noninvasive skin delivery.
• ICoN enables dermal-accessible NAD^+^ delivery in porcine and human skin explants.
• ICoN attenuates senescence signatures *in vitro* and human skin explants.
• Spatial transcriptomics maps ICoN-mediated reversal of aging hallmarks.
• ICoN extends lifespan and improves healthspan in *C. elegans*.

## Graphical abstract

**Figure:**
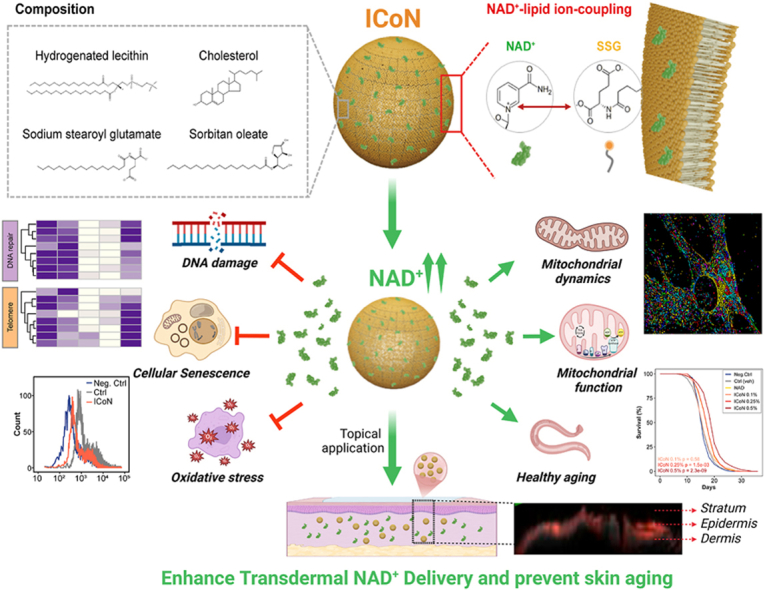

## 1 Introduction

Nicotinamide adenine dinucleotide (NAD^+^) is a pivotal molecule that regulates cellular metabolism and aging through diverse biochemical pathways [1,2]. As a coenzyme, NAD^+^ participates in oxidative phosphorylation, β-oxidation, glycolysis, intracellular calcium signaling, and gene expression regulation. As a substrate, it is consumed by poly(ADP-ribose) polymerases (PARPs) for DNA repair and by sirtuins for protein deacetylation [[3], [4], [5], [6], [7]]. Given its role in maintaining mitochondrial function and cellular homeostasis, preserving NAD^+^ levels is crucial for delaying aging-related decline [8,9]. However, NAD^+^ levels decline with age due to reduced nicotinamide phosphoribosyltransferase (NAMPT) expression, increased cluster of differentiation 38 (CD38) activity in immune cells, and PARP1 overactivation [2,[10], [11], [12], [13]]. This depletion impairs sirtuin activity, disrupting mitochondrial biogenesis and exacerbating oxidative stress, DNA damage, and metabolic dysfunction [14,15]. Thus, replenishing NAD^+^ is a promising strategy to counteract aging and metabolic disorders [16,17]. Despite its biological importance, NAD^+^ has a short intracellular half-life (∼1 h) and limited membrane permeability due to its positive charge and large molecular size [18]. As a result, supplementation relies on precursors like nicotinamide riboside (NR) and nicotinamide mononucleotide (NMN), which have demonstrated only modest NAD^+^ restoration in clinical and cellular studies [[19], [20], [21]]. Thus, a more effective delivery strategy is needed to enhance NAD^+^ bioavailability and stability.

Various delivery systems, including microneedles, polyelectrolyte complexes, lipid nanoparticles, and liposomes, have been explored for NAD^+^ administration [[22], [23], [24], [25]]. However, the skin imposes unique delivery challenges. Skin is composed of an avascular epidermis and a vascularized but poorly perfused dermis, making NAD^+^ replenishment difficult to achieve via systemic administration [[26], [27], [28]]. Moreover, the positively charged and hydrophilic nature of NAD^+^ hinders passive transport across biological membranes [29], and conventional oral or injectable routes rarely achieve pharmacologically meaningful concentrations inside dermal fibroblasts. Thus, the challenge of NAD^+^ restoration in skin is not purely biochemical, but fundamentally pharmacokinetic and anatomical, highlighting the need for strategies that directly address tissue-specific delivery constraints. Transfersomes, a class of elastic lipid vesicles, have shown promise in enhancing skin penetration by deforming through the stratum corneum [[30], [31], [32]]. Despite their advantages, conventional transfersomes using unsaturated lipids are prone to oxidation and require frozen storage, limiting their practical application [33]. Additionally, toxic solvents used in traditional thin-film hydration methods pose concerns for therapeutic applications [34].

Here, we demonstrate a novel ionically assembled NAD^+^ transfersome complex, termed Ion-Coupled NAD^+^ Transferosome (ICoN), designed to enhance NAD^+^ stability and enable transdermal delivery. This system electrostatically anchors NAD^+^ onto anionic lipid vesicles, reinforced by divalent fatty acids to strengthen molecular binding and improve vesicle stability. By enabling efficient and localized NAD^+^ replenishment within the skin, ICoN alleviates senescence and presents a promising transdermal strategy for skin aging.

## 2 Results

### 2.1 Preparation and Physicochemical Characterization of ICoN

**Fig. 1:**
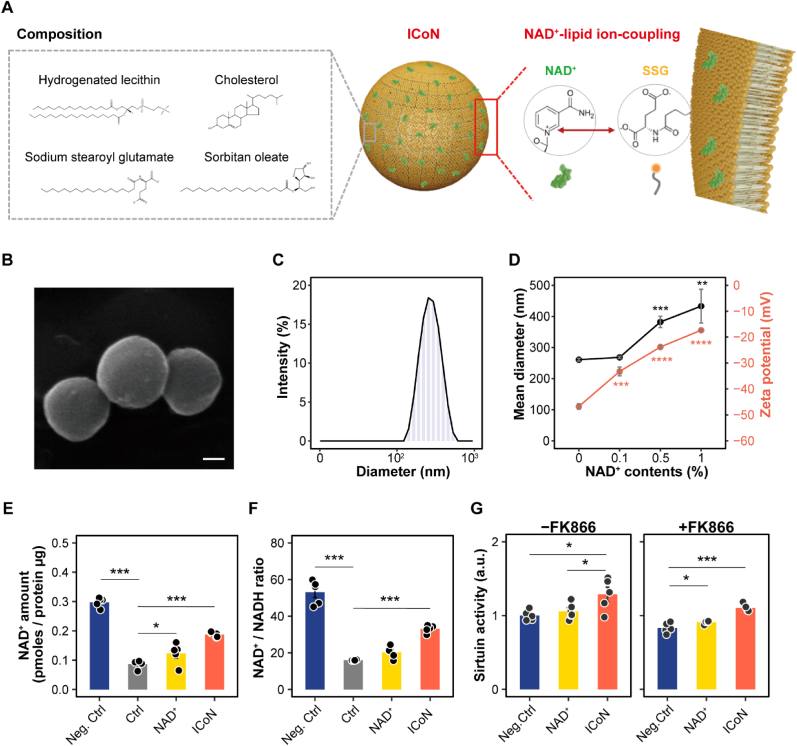
**Preparation and Physicochemical Characterization of ICoN**. (**A**) Schematic representation of ICoN (Ion-Coupled NAD^+^) formulation. Liposomes were prepared using hydrogenated lecithin, cholesterol, sodium stearoyl glutamate (SSG), and sorbitan oleate, where NAD^+^ was incorporated via ion coupling with SSG, ensuring stable entrapment within the lipid bilayer. (**B**) Scanning electron microscopy (SEM) image of ICoN liposomes. The nanoparticles appear spherical and well-formed, indicating a stable liposomal structure. Scale bar = 100 nm. (**C**) Size distribution analysis of ICoN nanoparticles measured by dynamic light scattering (DLS). The size distribution indicates that most particles fall within the 100-1000 nm range, with an inset showing a photograph of the ICoN suspension. (**D**) Effect of NAD^+^ incorporation on ICoN particle size (left y-axis, black line) and zeta potential (right y-axis, red line) (PDI = 0.155 ± 0.047, 0% NAD^+^; 0.164 ± 0.023, 0.1% NAD^+^; 0.174 ± 0.004, 0.5% NAD^+^; 0.182 ± 0.032, 1% NAD^+^) (n = 3, biological replicates). (**E, F**) Quantification of intracellular NAD^+^ levels (E) and NAD^+^/NADH ratio (F) in cells treated with ICoN (n = 5, biological replicates). (**G**) Sirtuin activation assay in cells treated with ICoN under normal conditions (-FK866) and under NAD^+^ depletion (+FK866) (n = 5, biological replicates). (For interpretation of the references to color in this figure legend, the reader is referred to the Web version of this article.)

NAD^+^ supplementation holds potential for ameliorating age-related and metabolic disorders, but its efficacy is constrained by rapid degradation, limited uptake, and poor stability. To overcome these limitations, a novel transdermal NAD^+^ delivery system, ICoN, was developed and characterized. ICoN was synthesized using hydrogenated lecithin, cholesterol, sodium stearoyl glutamate (SSG), and sorbitan oleate, with NAD^+^ ionically coupled to SSG, ensuring its stable integration within the lipid bilayer (Fig. 1A). Scanning electron microscopy (SEM) imaging confirmed the formation of well-defined, spherical nanoparticles, demonstrating the structural integrity of the complex (Fig. 1B). Dynamic light scattering (DLS) analysis showed a mean hydrodynamic diameter of ICoN in the nanoscale range, with a polydispersity index (PDI) of 0.164 ± 0.023, indicating acceptable colloidal homogeneity (Fig. 1C). To further characterize the particle size distribution, nanoparticle tracking analysis (NTA) was additionally performed, revealing that the majority of ICoN particles were distributed within the 50-200 nm range (Fig. S1). These data support that ICoN is composed predominantly of a relatively uniform vesicle population. The loading efficiency of NAD^+^ within the transfersome system was 80.8 ± 0.17%, indicating effective incorporation into the vesicular formulation. To assess the influence of NAD^+^ incorporation on particle characteristics, particle size and zeta potential were evaluated (Fig. 1D). Consistent with NAD^+^ intercalation into the vesicular structure, increasing NAD^+^ loading resulted in gradual enlargement of mean particle diameter. Although the zeta potential became moderately less negative, the electrostatic repulsion remained sufficient to preserve colloidal stability. A modest increase in PDI was observed with increasing NAD^+^ content. However, the values remained within an acceptable range, indicating that the overall size distribution was largely preserved and that NAD^+^ incorporation did not compromise vesicular integrity.

To further elucidate the molecular basis of NAD^+^ incorporation into the vesicular system, fluorescence resonance energy transfer (FRET) and Fourier-transform infrared (FTIR) spectroscopy were performed [35,36] (Fig. S2). FRET analysis revealed a donor-acceptor distance of approximately 5.4 nm (FRET efficiency: 40.9%) between NAD^+^ and the liposomal surface, indicating close spatial proximity and supporting molecular-level association between the two components. Complementary FTIR analysis showed that upon incorporation of NAD^+^, the characteristic ∼1713 cm^−1^ band of SSG diminished, accompanied by alterations in the ∼1560 cm^−1^ region, suggesting changes in the ionization state and local chemical environment of the carboxyl groups. These spectral features indicate that the presence of NAD^+^ perturbs the original carboxyl group interactions within SSG, consistent with a reorganization of intermolecular interactions at the vesicular interface. These findings support the presence of electrostatic and hydrogen-bonding interactions between NAD^+^ and SSG, providing experimental evidence for the proposed ion-coupling mechanism.

To further assess formulation robustness, physicochemical responses to environmental stress were examined by exposing ICoN to elevated temperatures. Notably, NAD^+^ retention was markedly improved in ICoN formulations, with significantly higher residual NAD^+^ content at elevated temperatures (40 °C: 60%, 50 °C: 50%) compared to free NAD^+^ solutions (40 °C: 10%, 50 °C: undetectable) (data not shown), indicating that the ion-coupling strategy effectively stabilizes NAD^+^ within lipid vesicles, preventing rapid degradation. Building on this observation, we next evaluated whether such resilience could be sustained over time. Hydrodynamic particle size and zeta potential were monitored over 4 weeks across diverse temperature conditions (Fig. S3). While a modest increase in PDI was observed at elevated temperatures, the values remained within an acceptable range, and no substantial changes in zeta potential were detected. These findings suggest that the overall physicochemical properties of ICoN are largely maintained during storage, supporting its stability.

To assess the efficiency of ICoN-mediated NAD^+^ delivery, NAD^+^ was fluorescently labeled with FITC to enable direct visualization (Fig. S4A). Fluorescent imaging of NAD^+^ revealed a pronounced intracellular accumulation following ICoN treatment compared with free NAD^+^, corroborated by quantitative assessments, including intracellular NAD^+^ content, fluorescence intensity, and a marked rightward shift in the FITC-A distribution in flow cytometry profiles (Fig. S4B–D). Notably, pharmacological inhibition of endocytosis did not substantially reduce ICoN-mediated NAD^+^ uptake (Fig. S4E), indicating translocation is largely independent of classical, energy-dependent endocytic routes. This suggests a direct membrane interaction mechanism of ICoN, which may offer distinct advantages in cellular states with compromised endocytotic capacity, such as metabolic impairment or senescence, thereby enabling NAD^+^ replenishment in otherwise refractory cellular states.

Although both 10 ppm and 20 ppm ICoN improved NAD^+^ delivery, 10 ppm was selected for downstream assessments to ensure metabolic relevance while avoiding supraphysiological dosing. Accordingly, intracellular NAD^+^ levels and NAD^+^/NADH ratio were quantified following treatment (Fig. 1E–F). ICoN significantly elevated NAD^+^ levels and improved redox balance compared with free NAD^+^. To investigate its impact on NAD^+^-dependent pathways, sirtuin activation was evaluated under both normal conditions and severe NAD^+^ depletion induced by FK866, a potent NAMPT inhibitor (Fig. 1G). Under normal conditions, ICoN facilitated greater sirtuin activation compared to free NAD^+^, while under FK866-induced NAD^+^ depletion, ICoN restored sirtuin activity, demonstrating its ability to sustain NAD^+^-dependent metabolic functions even under metabolic stress.

To determine whether ICoN maintains immunological compatibility in skin during NAD^+^ delivery, nitric oxide release in RAW264.7 macrophages and β-hexosaminidase–mediated degranulation in RBL-2H3 were assessed (Fig. S5). Neither free NAD^+^ nor ICoN induced a significant increase in either readout, indicating the biosafety for topical or transdermal application.

Together, these findings establish ICoN as a robust NAD^+^ delivery system, offering enhanced stability, bioavailability, and functional efficacy.

### 2.2 Unbiased Gene Set Enrichment Analysis Reveals Transcriptional Patterns Associated with ICoN treatment

To determine whether NAD^+^ depletion represents a shared feature across distinct aging-associated stress conditions, intracellular NAD^+^ levels were quantified in human skin fibroblasts subjected to UV irradiation, oxidative stress, and chronological aging (Fig. S6). In all cases, NAD^+^ levels were consistently reduced, indicating that diverse aging-related stressors converge on a decline in cellular NAD^+^ availability. Based on this observation, the FK866 model was employed as a reductionist approach to interrogate NAD^+^-dependent transcriptional regulation at high resolution.

**Fig. 2:**
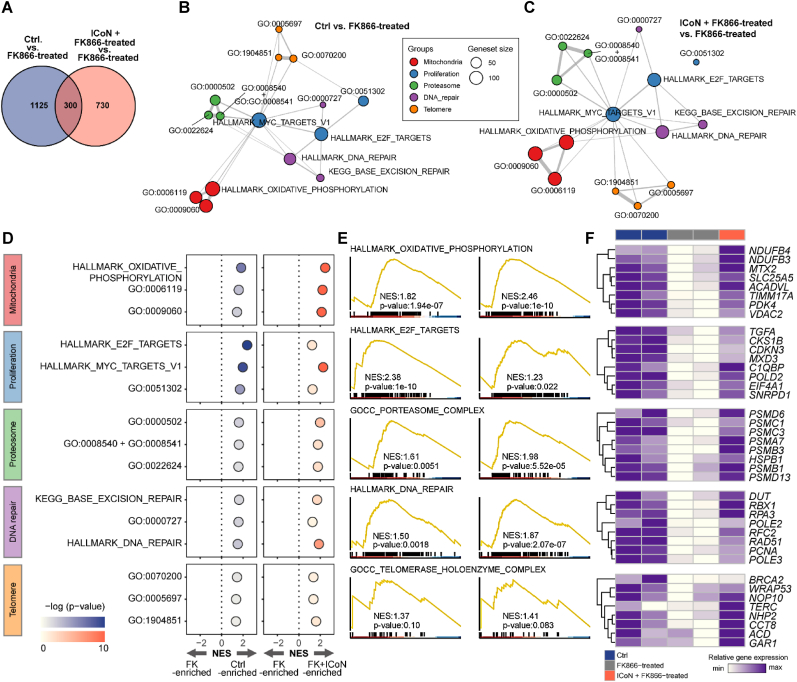
**Unbiased Gene Set Enrichment Analysis Reveals Transcriptional Patterns Associated with ICoN Treatment.** (**A**) Venn diagram showing the number of differentially expressed genes (DEGs) between Ctrl vs. FK866-treated (blue) and ICoN + FK866-treated (red) vs. FK866-treated. (**B, C**) Network diagrams of significantly enriched gene sets. Enriched pathways in the FK866-treated vs. control comparison (B), highlighting mitochondrial function (red), proliferation (blue), proteasome activity (green), DNA repair (purple), and telomere maintenance (orange). Enriched pathways in the ICoN + FK866-treated vs. FK866-treated comparison (C), showing similar clusters, suggesting ICoN restores NAD^+^-related pathways. Node size represents the number of genes in each pathway, and edges indicate functional relationships. (**D**) Bubble plot showing the enrichment of key pathways across the three comparisons: FK866-treated vs. control (FK-enriched), control vs. FK866-treated (Ctrl-enriched), and ICoN + FK866-treated vs. FK866-treated (FK + ICoN-enriched). Dot color represents statistical significance (-log p-value), while dot size represents enrichment magnitude. (**E**) Gene Set Enrichment Analysis (GSEA) plots for selected pathways. Normalized enrichment scores (NES) and p-values indicate the degree of pathway activation or suppression. ICoN restores oxidative phosphorylation, cell cycle regulation (E2F targets), proteasome activity, DNA repair, and telomere maintenance, suggesting recovery of NAD^+^-dependent functions. (**F**) Heatmap of selected genes from enriched pathways. Gene expression levels are shown across control (blue), FK866-treated (gray), and ICoN + FK866-treated (red) conditions. RNA-seq analysis was performed using a limited number of samples per condition (Ctrl, n = 2; FK866-treated, n = 2; ICoN + FK866-treated, n = 1). (For interpretation of the references to color in this figure legend, the reader is referred to the Web version of this article.)

To explore the molecular features associated with ICoN treatment, transcriptomic profiling was performed using Gene Set Enrichment Analysis (GSEA) (Fig. 2). RNA sequencing data were obtained from three experimental conditions: control (Ctrl), NAD^+^-depleted cells treated with FK866 (FK866-treated), and FK866-treated cells supplemented with ICoN (ICoN + FK866-treated). Comparisons were performed between Ctrl vs. FK866-treated and ICoN + FK866-treated vs. FK866-treated to examine transcriptional alterations associated with NAD^+^ depletion and ICoN supplementation.

GSEA identified 1425 significantly dysregulated gene sets in FK866-treated cells compared to control, while 1030 gene sets were differentially expressed in ICoN + FK866-treated cells relative to FK866-treated cells (Fig. 2A). Among these, 300 gene sets were shared between the two comparisons, suggesting partial overlap in transcriptional patterns altered by FK866 and those associated with ICoN treatment. These gene sets primarily involved mitochondrial function, cell cycle progression, proteostasis, DNA repair, and telomere maintenance, all of which have been previously linked to NAD^+^-dependent biological processes. Pathway-enrichment analysis revealed that ICoN treatment was associated with increased representation of gene sets linked to mitochondrial oxidative phosphorylation, cell cycle regulation (E2F and MYC targets), proteasome-related gene expression, and DNA repair, indicating that ICoN counteracts metabolic and genomic stress (Fig. 2B–C). These findings reflect transcriptional enrichment patterns rather than direct functional measurements, and imply cellular responses to altered NAD^+^ availability. Consistent with this interpretation, gene sets related to mitochondrial oxidative phosphorylation (OXPHOS), proteasomal components, and DNA repair factors showed higher normalized enrichment scores (NES) in the ICoN-treated condition (Fig. 2D–E). Representative GSEA plots illustrate these enrichment patterns across selected pathways (Fig. 2F).

Together, these analyses suggest that ICoN treatment is associated with partial reversal of transcriptional alterations induced by NAD^+^ depletion, particularly in pathways related to metabolic function and genomic maintenance.

### 2.3 ICoN Protects Against DNA Damage, Oxidative Stress, and Cellular Senescence *in Vitro*

**Fig. 3:**
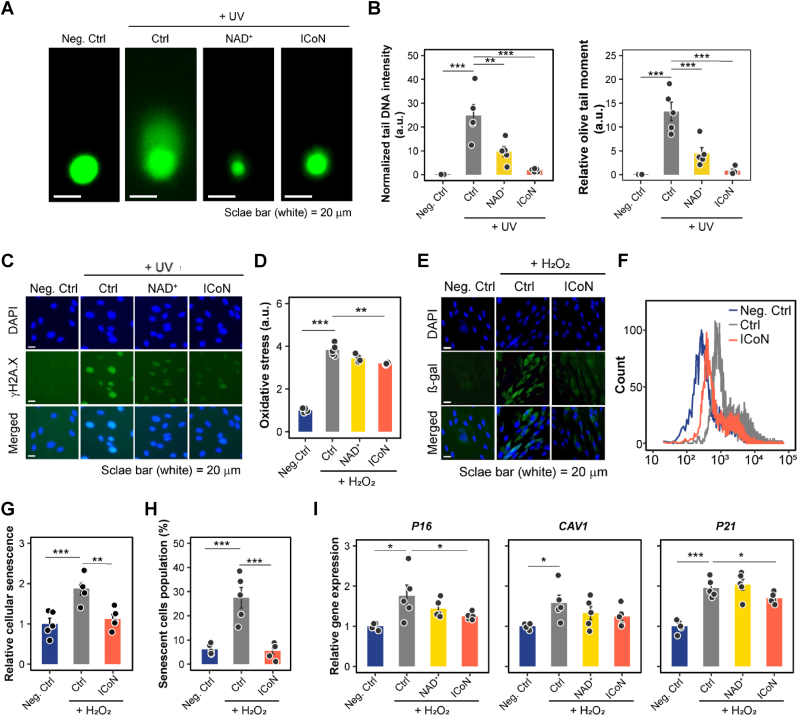
**ICoN Protects Against DNA Damage, Oxidative Stress, and Cellular Senescence *in Vitro*.** (**A**) Comet assay images of cells exposed to UV radiation and treated with negative control (Neg. Ctrl, without UV radiation), vehicle-treated control (Ctrl), NAD^+^, or ICoN. Scale bar = 20 μm. (**B**) Quantification of comet assay results. Left: Normalized tail DNA intensity. Right: Relative olive tail moment (n = 5, biological replicates). (**C**) Immunofluorescence staining of γH2A.X, in UV-exposed cells. Scale bar = 20 μm. (**D**) Intracellular reactive oxygen species (ROS) levels measured after H_2_O_2_ exposure (n = 6, biological replicates). (**E**) β-galactosidase (β-gal) staining of senescent cells following H_2_O_2_-induced oxidative stress. Scale bar = 20 μm. (**F-H**) Flow cytometry analysis of senescence-associated fluorescence intensity. The mean fluorescence intensity (MFI) was determined by flow cytometry from 10,000 cells per sample. (F) Quantification of senescent cells following H_2_O_2_ exposure. ICoN-treated cells show a significant reduction in relative cellular senescence intensity (g) and senescent cell population (h) (n = 5, biological replicates). (**I**) mRNA analysis of senescence-associated secretory phenotype markers, including *P16*, *CAV1*, and *P21* (n = 5, biological replicates).

To functionally assess these transcriptional patterns, we next examined the effects of ICoN on DNA damage, oxidative stress, and cellular senescence. A comet assay was performed following ultraviolet (UV)-induced genotoxic stress to visualize DNA damage. Cells exposed to UV exhibited significant DNA fragmentation, as indicated by increased DNA tail intensity and olive tail moment (Fig. 3A–B). However, ICoN supplementation significantly reduced DNA fragmentation compared to untreated and free NAD^+^-treated cells, demonstrating its protective role in preserving genomic integrity. Furthermore, ICoN-treated cells displayed greater DNA repair efficiency, suggesting that its sustained NAD^+^ bioavailability enhances the activity of DNA repair pathways.

To further assess NAD^+^-dependent DNA repair, γH2A.X immunofluorescence staining was performed to detect UV-induced DNA double-strand breaks. Compared to the untreated control group, cells subjected to UV irradiation exhibited significantly higher γH2A.X fluorescence intensity, indicating an accumulation of DNA damage. ICoN-treated cells displayed a marked reduction in γH2A.X signals compared to vehicle- and free NAD^+^-treated cells, further confirming its role in mitigating DNA damage and enhancing repair efficiency (Fig. 3C).

Next, oxidative stress levels were assessed using the DCFDA assay to measure reactive oxygen species (ROS) accumulation following H_2_O_2_ treatment. Control cells exhibited a significant increase in oxidative stress, whereas ICoN supplementation effectively suppressed ROS accumulation (Fig. 3D), confirming its role in NAD^+^-dependent antioxidant defense mechanisms. To further evaluate ICoN's effect on oxidative stress-induced cellular senescence, β-galactosidase (β-gal) staining was performed. Upon H_2_O_2_ treatment, β-gal activity markedly increased in vehicle-treated control (Ctrl) cells compared to the negative control (Neg. Ctrl), indicating a rise in cellular senescence features. However, co-treatment with ICoN significantly reduced β-gal-positive staining, suggesting that ICoN effectively mitigates senescence-associated β-gal (SA-β-gal) activation under oxidative stress conditions (Fig. 3E). Flow cytometry analysis further corroborated these findings, demonstrating a leftward shift in fluorescence intensity in ICoN-treated cells, suggesting lower expression of senescence markers compared to control and free NAD^+^-treated groups (Fig. 3F–H).

Analysis of gene expression confirmed that senescence-associated markers (*P16*, *CAV1*, and *P21*) were significantly upregulated following H_2_O_2_-induced oxidative stress, whereas ICoN supplementation effectively suppressed their expression (Fig. 3I). Additionally, ICoN reduced the expression of pro-inflammatory senescence-associated secretory phenotype markers, including *MMP9*, *MCP2*, *IL1B*, *IL6*, *IL8*, *PAI2*, and *CXCL1*, further supporting its anti-inflammatory and senescence-mitigating effects (Fig. S7).

Together, these findings demonstrate that ICoN effectively mitigates oxidative stress and UV-induced DNA damage by reducing DNA fragmentation, suppressing ROS accumulation, and inhibiting senescence-associated inflammation.

### 2.4 ICoN Modulates Mitochondrial Dynamics and Sustains Bioenergetic Function under Senescent States *in Vitro*

**Fig. 4:**
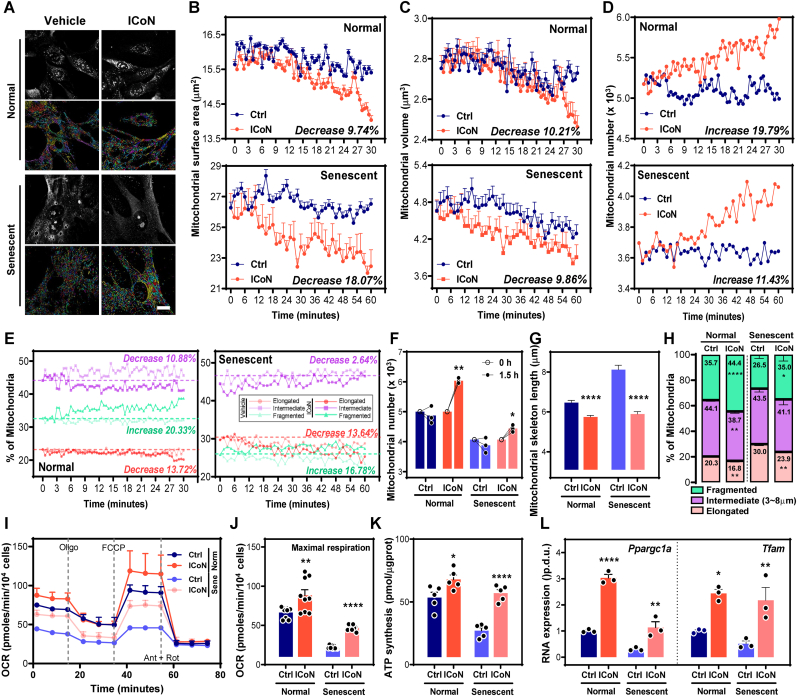
**ICoN Modulates Mitochondrial Dynamics and Sustains Bioenergetic Function under Senescent States *in Vitro*.** (**A**) Representative label-free, real-time images of mitochondrial networks illustrating mitochondrial dynamics in normal and senescent cells treated with either vehicle or ICoN. Scale bar = 20 μm. (**B-D**) Quantification of mitochondrial dynamics over time. (B) Mitochondrial surface area, (C) mitochondrial volume, and (D) mitochondrial number in normal (upper) and senescent (lower) cells. (n ranges 3000-6000, biological replicates). (**E**) Time-course analysis of mitochondrial dynamics in normal and senescent cells, by calculating the percentage of mitochondria exhibiting morphological features based on skeleton length: elongation (≥8 μm), intermediate (3∼8 μm), and fragmentation (<3 μm). ICoN treatment enhances mitochondrial fragmentation while reducing elongation compared to vehicle-treated cells, in both normal and senescent cells. Initial indexes for ICoN-treated cells at Time 0 were normalized to those of the vehicle-treated group for comparative analysis. Variations annotated in each panel indicate intragroup comparisons at the endpoint of observation. (**F**) Alteration in mitochondrial number in vehicle- or ICoN-treated cells over 1.5 h. ICoN treatment facilitates mitochondrial biogenesis, in both normal and senescent cells. Initial mitochondrial numbers for the ICoN-treated group (untreated baseline) were normalized to the vehicle-treated group for comparative analysis (n = 3, biological replicates). (**G**) Quantification of mitochondrial skeleton length in normal and senescent cells (n ranges 7500-11000, biological replicates). (**H**) Classification of mitochondrial morphology across experimental conditions. Senescent cells show higher proportion of elongated mitochondria, whereas ICoN treatment increases mitochondria fragmentation in both normal and senescent cells (n = 3, biological replicates). (**I**) Oxygen consumption rate (OCR) analysis. Mitochondrial respiration was measured using extracellular flux analysis following oligomycin (Oligo), FCCP, antimycin A (Ant A), and rotenone (Rot) treatment. (**J**) Quantification of maximal respiration in panel I (n = 9, biological replicates). (**K**) ATP levels quantified by enzyme-linked immunosorbent assay (n = 5, biological replicates). (**L**) mRNA analysis of mitochondrial function-associated markers, including *Ppargc1a* and *Tfam* (n = 3, biological replicates).

Mitochondrial dynamics are essential for sustaining mitochondrial functional integrity, particularly under conditions where organelle integrity must be actively preserved. Cellular senescence is accompanied by persistently elongated mitochondrial networks and diminished renewal competence, which are indicative of a reduced but potentially recoverable capacity for structural remodeling [37,38]. To examine whether NAD^+^ modulation via ICoN could engage residual mitochondrial remodeling capacity in the senescent state, we employed AI-assisted, label-free 3D live-cell imaging (HT-X1) for unbiased and high-resolution quantification of multi-dimensional mitochondrial structural parameters in senescent fibroblasts (Fig. 4A).

These metrics, including mitochondrial number and individual mitochondrial morphometrics (length, surface area, volume), were automatically tracked over time to delineate potential alterations in mitochondrial organization following ICoN treatment. In normal fibroblasts, mitochondrial surface area and volume remained largely stable in the control group but showed a measurable reduction upon ICoN treatment (Fig. 4B–C, upper), revealing that mitochondrial architecture retains adaptive responsiveness to NAD^+^ modulation under homeostatic conditions. Notably, mitochondrial number increased substantially following ICoN treatment (Fig. 4D, upper), reflecting a redistribution into smaller mitochondrial units rather than a depletion of mitochondrial content. This pattern is consistent with a preparatory remodeling state rather than simple degeneration. A more gradual pattern was observed in senescent fibroblasts, where mitochondrial surface area and volume declined mildly in the control group yet more prominently following ICoN treatment (Fig. 4B–C, lower). Mitochondrial number increased selectively in the ICoN-treated group (Fig. 4D, lower), highlighting architectural redistribution rather than loss of mitochondrial mass, even within a metabolically compromised environment.

To further resolve the nature of this remodeling, mitochondria were classified into fragmented, intermediate, and elongated populations based on AI-defined morphological signatures (Fig. 4E–H). ICoN treatment consistently reduced elongated mitochondria while increasing fragmented populations in both normal and senescent cells, denoting a shift toward enhanced mitochondrial segmentation and architectural reorganization. Importantly, this transition was not accompanied by signs of organelle depletion; instead, the reduction in individual mitochondrial size occurred alongside an increase in mitochondrial number (Fig. 4F), substantiating a redistributed mitochondrial configuration rather than structural deterioration.

To determine whether this reorganizational shift carries functional significance, we next assessed mitochondrial bioenergetics. Given that fragmentation alone does not inherently signify dysfunction and may instead represent a transitional state of structural reorganization, we evaluated whether such architectural changes corresponded to metabolic enhancement. ICoN treatment significantly increased mitochondrial respiration in both normal and senescent conditions (Fig. 4I–J, Fig. S8), demonstrating that the reorganized mitochondrial population retained oxidative capacity rather than undergoing degenerative remodeling. Consistently, intracellular ATP levels were elevated following ICoN treatment (Fig. 4K), corroborating improved energetic competence within the reshaped mitochondrial network. In parallel, transcriptional analysis revealed upregulation of key regulators associated with mitochondrial biogenesis and functional maintenance (Fig. 4L), supporting activation of renewal-related programs rather than structural decline.

Together, these observations demonstrate that ICoN treatment modifies mitochondrial architecture while preserving and enhancing mitochondrial dynamics in both normal and senescent cells.

### 2.5 ICoN Preserves Mitochondrial Function Under NAD^+^ Depletion *in Vitro*

**Fig. 5:**
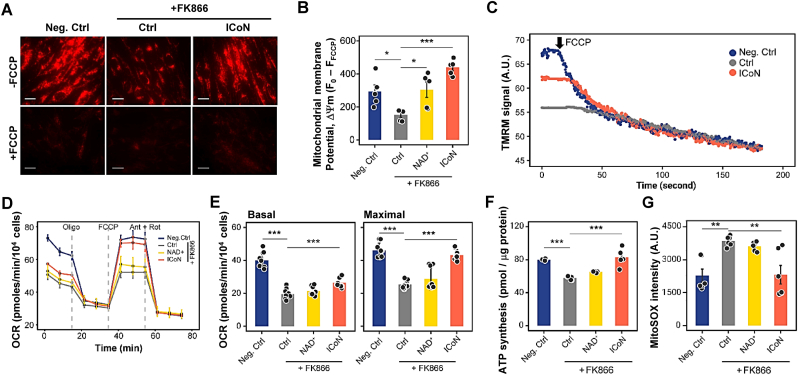
**ICoN Preserves Mitochondrial Function Under NAD**^**+**^**Depletion *in Vitro*.** (**A-C**) Mitochondrial membrane potential (ΔΨm) restoration. (A) Representative TMRM fluorescence images of negative control, FK866-treated, and ICoN and FK866 co-treated cells. Scale bar = 20 μm. (B) End-point quantification of ΔΨm calculated relative to FCCP baseline using the equation: ΔΨm = (F_treatment_ - F_FCCP_) (n = 5, biological replicates). (C) Real-time fluorescence kinetics of TMRM signal following FCCP challenge. Values represent absolute fluorescence intensity (A.U.). (**D-E**) OCR analysis with quantification of basal and maximal respiration (n = 9, biological replicates). (**F**) Quantification of ATP level (n = 5, biological replicates). (**G**) Quantification of MitoSOX fluorescence intensity reflecting mitochondrial oxidative stress (n = 5, biological replicates). The MFI was determined by flow cytometry from 10,000 cells per sample.

To assess whether the ICoN-mediated mitochondrial improvements identified in senescent cells are preserved across distinct metabolic stress contexts, we examined key bioenergetic parameters in a model of FK866-induced NAD^+^ depletion, which imposes direct metabolic constraint. TMRM fluorescence intensity, an indicator of mitochondrial membrane potential, was significantly reduced in FK866-treated cells compared to negative controls (Fig. 5A–B). ICoN supplementation effectively restored ΔΨm, indicating improved mitochondrial function and membrane integrity even under acute NAD^+^ depletion. Importantly, the mitochondrial uncoupler Carbonyl cyanide p-trifluoromethoxyphenylhydrazone (FCCP) completely abolished this effect, confirming that the observed increase in ΔΨm represented a genuine stabilization of mitochondrial membrane potential (Fig. 5C).

To further evaluate whether ICoN maintains mitochondrial function under acute metabolic constraint, oxidative phosphorylation was next examined. NAD^+^ deficiency markedly impaired mitochondrial respiration, which was reversed by ICoN supplementation (Fig. 5D–E), highlighting its preserved respiratory capacity in this acute metabolic stress model. Similarly, ATP synthesis, compromised by NAD^+^ depletion, was significantly restored following ICoN treatment, further supporting its ability to sustain mitochondrial function (Fig. 5F). Mitochondrial oxidative stress was evaluated using MitoSOX fluorescence, revealing a pronounced increase in ROS levels following FK866 exposure; however, ICoN significantly attenuated ROS accumulation (Fig. 5G, Fig. S9), suggesting that preservation of mitochondrial function is accompanied by enhanced redox balance under NAD^+^ depletion.

Together, these data demonstrate that ICoN mitigates the functional consequences of acute NAD^+^ depletion by sustaining oxidative phosphorylation, restoring ATP production, and limiting mitochondrial oxidative stress, complementing the structural and dynamic remodeling observed in senescent cells.

### 2.6 ICoN Promotes Cell Migration, ECM-associated Gene Expression, and Cell Proliferation Under NAD^+^ Depletion *in Vitro*

**Fig. 6:**
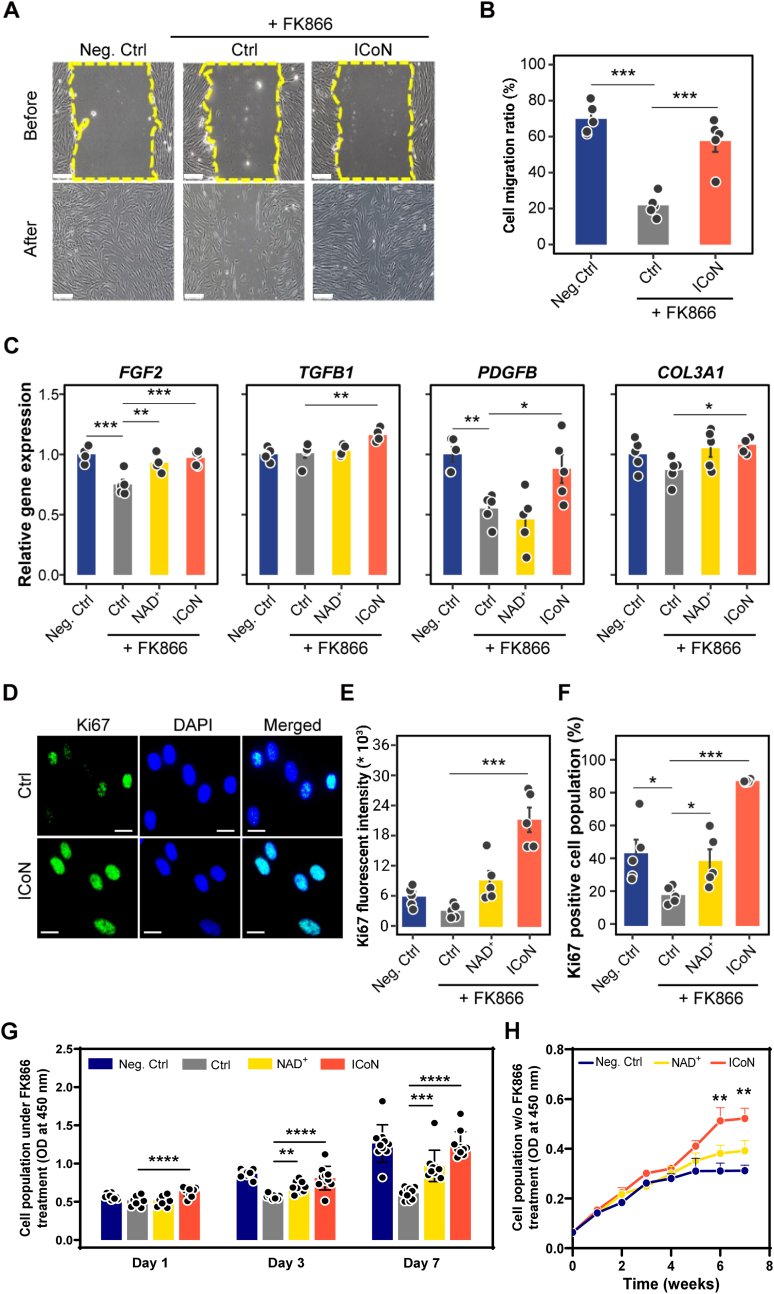
**ICoN Promotes Cell Migration, ECM-associated Gene Expression, and Cell Proliferation Under NAD**^**+**^**Depletion *in Vitro*.** (**A, B**) Cell migration assay. (A) Representative phase-contrast images show the wound area before and after migration. The dashed yellow lines indicate the initial wound boundary. Scale bar = 200 μm. (B) Quantification of cell migration ratio (n = 5, biological replicates). (**C**) mRNA analysis of ECM remodeling and growth factor genes, including *FGF2* (fibroblast growth factor 2), *TGFB1* (transforming growth factor-beta 1), *PDGFB* (platelet-derived growth factor B) and *COL3A1* (collagen type III) (n = 5, biological replicates). (**D-F**) Ki67 immunofluorescence staining. Representative images of Ki67 (green) and nuclear counterstaining with DAPI (blue). Scale bar = 20 μm (D). Quantification of Ki67 fluorescence intensity (E) and Ki67-positive cell population (F) (n = 5, biological replicates). A total of 10,000 cells were analyzed using flow cytometer. (**G**) Quantification of viable cell numbers under FK866-induced NAD^+^ depletion on days 1, 3, and 7 (n = 10, biological replicates). (**H**) Long-term quantification of viable cell numbers in the absence of FK866 from weeks 1 to 7 (n = 10, biological replicates). (For interpretation of the references to color in this figure legend, the reader is referred to the Web version of this article.)

Building upon previous findings demonstrating ICoN's role in preserving mitochondrial homeostasis and bioenergetics, its effects on cellular functions related to tissue repair were further examined. Specifically, cell migration, extracellular matrix (ECM)-associated gene expression, and cell proliferation were evaluated under NAD^+^ depletion to determine whether ICoN mitigates the detrimental effects of metabolic stress on these processes. To assess ICoN's influence on cell migration, a scratch assay was conducted under NAD^+^ depletion-induced cellular damage (Fig. 6A). Representative phase-contrast images captured before and after wound closure revealed that NAD^+^ depletion by FK866 markedly delayed wound closure in control cells, whereas ICoN-treated cells exhibited accelerated cell migration, underscoring its promise in promoting tissue repair (Fig. 6B).

To characterize ECM-related molecular responses, gene expression analysis revealed that ICoN treatment significantly upregulated fibroblast growth factor 2 (*FGF2*), transforming growth factor-beta 1 (*TGFB1*), platelet-derived growth factor B (*PDGFB*), and collagen type III (*COL3A1*) compared to NAD^+^-depleted cells (Ctrl). Notably, only *FGF2* and *PDGFB* were significantly downregulated by NAD ^+^ depletion (Fig. 6C).

To further evaluate ICoN's effect on cellular proliferation, Ki67 immunofluorescence staining was performed. ICoN-treated cells exhibited a substantial increase in Ki67-positive nuclei compared to controls, indicating enhanced proliferative activity (Fig. 6D). Quantitative analysis of Ki67 fluorescence intensity (Fig. 6E) and the proportion of Ki67-positive cells (Fig. 6F) further confirmed that ICoN supplementation effectively counteracted NAD^+^ depletion-induced proliferative arrest. As Ki67 reflects the proportion of cells actively engaged in the cell cycle rather than cumulative population growth, population‐level proliferation assays were performed to evaluate replicative output beyond cell cycle status. ICoN supplementation led to a progressive increase in viable cell numbers under FK866-induced NAD^+^ depletion during short-term culture (Fig. 6G), indicating improved proliferative capacity even under metabolic constraint. In addition, long-term population kinetics assessed in the absence of FK866 demonstrated that ICoN extended proliferative longevity, with time to near-saturation increasing from 30.2 days (Neg. Ctrl) or 42.8 days (NAD^+^) to 54.9 days (ICoN), reflecting enhanced durability of population expansion under physiological conditions (Fig. 6H).

Together, these data suggest that ICoN supports cell migration and upregulates ECM-associated gene expression while preserving proliferative capacity under NAD^+^ depletion, implying its potential to support tissue repair in metabolically compromised environments.

### 2.7 ICoN Facilitates NAD^+^ Penetration in a 3D *Ex Vivo* Porcine Skin Explant Model

**Fig. 7:**
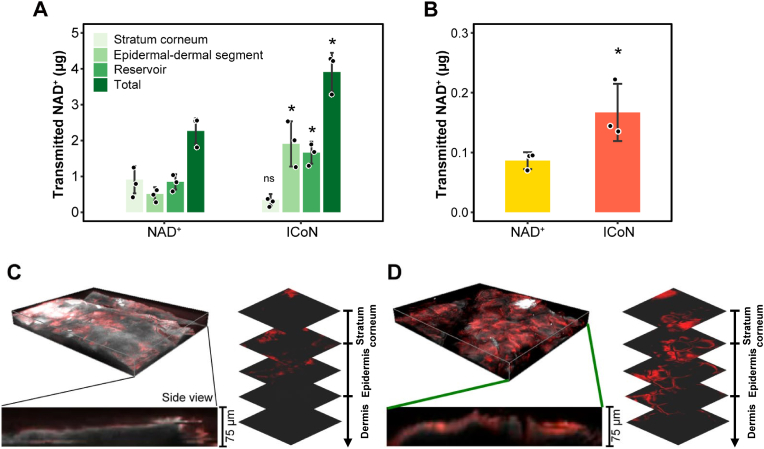
**ICoN Facilitates NAD**^**+**^**Penetration in a 3D *Ex Vivo* Porcine Skin Explant Model.** (**A**) Transdermal delivery of NAD^+^ and ICoN in Franz Cell Membrane (Porcine skin). Quantification of NAD^+^ penetration across different skin layers, including the stratum corneum (light green), skin tissue (green), reservoir (the receiver solution beneath the Franz cell membrane, dark green), and total absorption (darkest green) (n = 3, biological replicates) (**B**) Enhanced transdermal NAD^+^ delivery using ICoN in a topical formulation (n = 3, biological replicates). (**C-D**) Fluorescence imaging of transdermal NAD^+^ delivery. ICoN promotes enhanced transdermal penetration of NAD^+^ into the dermis layer. (For interpretation of the references to color in this figure legend, the reader is referred to the Web version of this article.)

Efficient transdermal delivery of NAD^+^ remains challenging due to its hydrophilic nature and limited passive transport across the stratum corneum. To evaluate delivery performance in a physiologically relevant context, a 3D *ex vivo* porcine skin explant model was employed, representing a structured barrier containing the stratum corneum, epidermis, and upper dermis. This setup allows assessment of NAD^+^ permeation across multiple skin compartments rather than monolayer uptake, providing greater biological relevance for topical evaluation. Topical application of free NAD^+^ or ICoN was followed by compartment-specific quantification of NAD^+^ in (i) the stratum corneum, (ii) the combined epidermal-dermal segment, and (iii) the receiver solution beneath the Franz cell membrane (referred to as the reservoir, Fig. 7A). Because enzymatically active separation or heat-induced separation of epidermal and dermal layers risks perturbing endogenous NAD^+^ turnover and degrading labile pyridine nucleotides, we opted to quantify NAD^+^ in full-thickness viable skin explants. The resulting measurements therefore represent a composite signal across extra- and intracellular compartments and all included layers, rather than a layer-resolved readout. ICoN-treated samples exhibited significantly higher NAD^+^ levels across all compartments compared to free NAD^+^, with prominent accumulation in the epidermal-dermal region. Detection of NAD^+^ in the reservoir further indicated successful trans-barrier passage, reflecting permeation beyond the superficial epidermis. To assess whether the delivery effect of ICoN is sustained within a formulation context, a topical cream was prepared either with ICoN or with free NAD^+^. ICoN consistently delivered higher levels of NAD^+^ into skin tissue (Fig. 7B), despite identical NAD^+^ concentrations across preparations.

To visualize the spatial distribution of NAD^+^ within the porcine skin explant, fluorescence imaging was performed (Fig. 7C–D). Free NAD^+^ remained predominantly confined to the stratum corneum, with minimal penetration into the epidermis. Conversely, ICoN facilitated clear NAD^+^ accumulation within the epidermis and extended into the dermis. Consistently, side-view imaging revealed vertical permeation across skin layers under ICoN treatment, indicating trans-barrier transport rather than superficial retention.

Together, these findings emphasize that ICoN enhances transdermal NAD^+^ transport across multiple skin compartments and retains its delivery efficacy even within a formulation matrix.

### 2.8 ICoN Facilitates NAD^+^ Delivery and Attenuates Senescence Signatures in an *Ex Vivo* Human Skin Explant

**Fig. 8:**
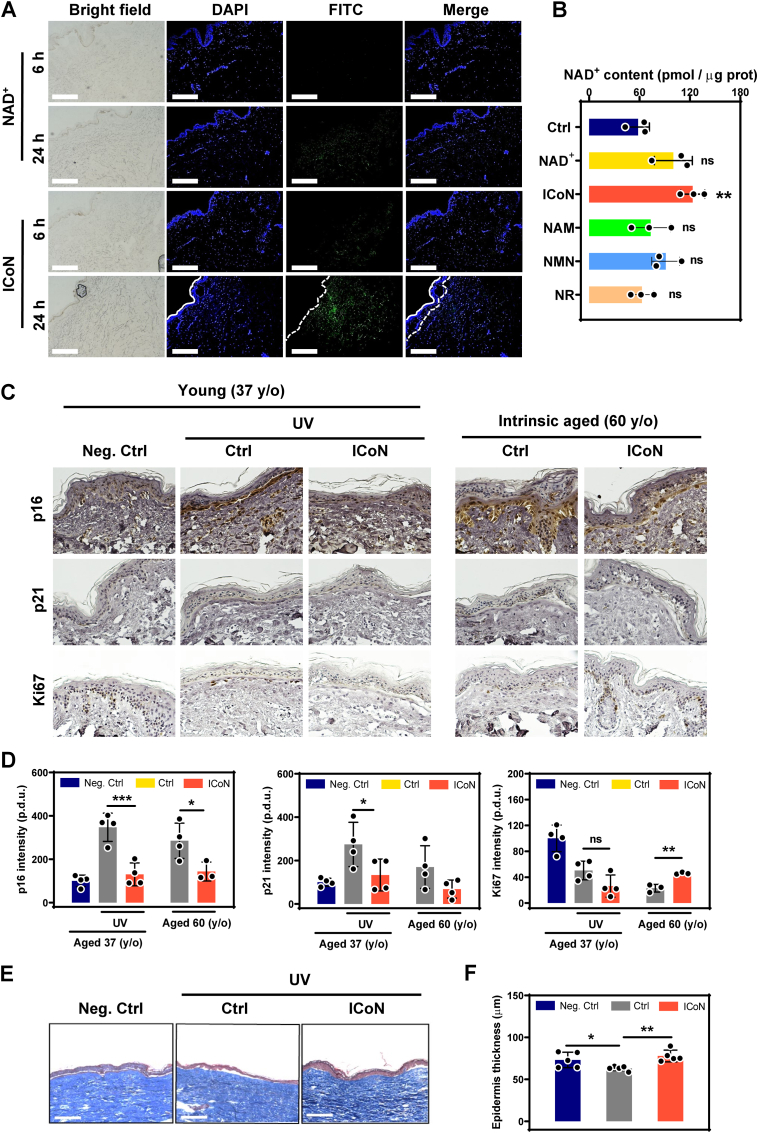
**ICoN Facilitates NAD**^+^**Delivery and Attenuates Senescence Signatures in a 3D*****Ex Vivo*****Human Skin Explant Model.** (**A**) Representative bright-field and confocal fluorescence images of *ex vivo* human skin explants following topical application of FITC-labeled NAD^+^ or ICoN for 6 or 24 h. Nuclei were counterstained with DAPI. Scare bar = 275 μm. (**B**) Quantification of intratissue NAD^+^ content in human skin explants treated with free NAD^+^, ICoN, or NAD^+^ precursors (NAM, NMN, NR) (compared to Ctrl, n = 3, biological replicates (explants)). (**C-D**) Representative immunohistochemical staining and quantification of senescence markers p16 and p21, and the proliferation marker Ki67, in human skin explants derived from a young donor subjected to UV-induced photoaging (37 y/o) and an intrin sically aged donor (60 y/o), with or without ICoN treatment (n = 4, biological replicates). Expression of senescent markers and Ki67 in human skin explants exposed to UV irradiation every two days, followed by topical application of ICoN or vehicle every three days for 12 days. IHC-positive areas were quantified from three tissue sections per biological replicate (technical replicates), and the mean value was used for statistical analysis. Scale bar = 200 μm. (**E-F**) Histological analysis via Masson's trichrome staining and quantification of epidermal thickness (F) (n = 5, biological replicates).

To evaluate the performance of ICoN in a more physiologically representative human tissue context, we next employed an *ex vivo* human skin explant model derived from intrinsically aged donors. FITC-tagged NAD^+^ and ICoN were topically applied for 6 or 24 h, followed by high-resolution confocal imaging to assess spatial distribution across skin layers. Free NAD^+^ exhibited minimal penetration and remained largely restricted to the stratum corneum, whereas ICoN achieved clear access to dermis, demonstrating efficient trans-barrier transport into metabolically active fibroblast-rich regions (Fig. 8A). The fluorescence signal further intensified at 24 h, suggesting sustained tissue permeation rather than transient surface deposition.

To compare ICoN with common strategies for enhancing NAD^+^ levels in skin tissue, explants were treated with free NAD^+^, ICoN, or widely used NAD^+^ precursors (NR, NMN, NAM) under comparable conditions (1%, w/v). ICoN was associated with a greater increase in intratissue NAD^+^ levels compared to free NAD^+^ and precursors (Fig. 8B). Given that the treatments were not applied at equimolar concentrations, these results should be interpreted as a comparative outcome rather than a direct assessment of relative efficacy.

While intrinsic aging compromises NAD^+^ homeostasis, chronic UV exposure is a major extrinsic driver of cutaneous aging, leading to DNA damage, oxidative stress, inflammation, and progressive epidermal thinning [39]. To investigate whether ICoN supports tissue resilience under both aging contexts, human skin explants were obtained from an intrinsic-aged donor (60 y/o) and a UV-induced photoaging model (37 y/o). ICoN treatment reduced p16 and p21 expression while increasing Ki67 positivity in both models (Fig. 8C–D), indicating mitigation of senescence signaling and restoration of proliferative competence. Complementary histological analyses using photoaging model revealed partial recovery of epidermal thickness following ICoN treatment in UV-stressed tissue (Fig. 8E–F), emphasizing a regenerative response that aligns with molecular reversal of senescence.

Together, these findings demonstrate that ICoN enables functional NAD^+^ access to metabolically active regions of human skin and counteracts senescence-associated molecular and structural alterations, supporting its potential as a targeted transdermal NAD^+^ delivery strategy for aging-associated tissue decline.

### 2.9 ICoN Attenuates UV-Induced Histological and Spatial Transcriptomic Features in *Ex Vivo* Aged Human Skin

**Fig. 9:**
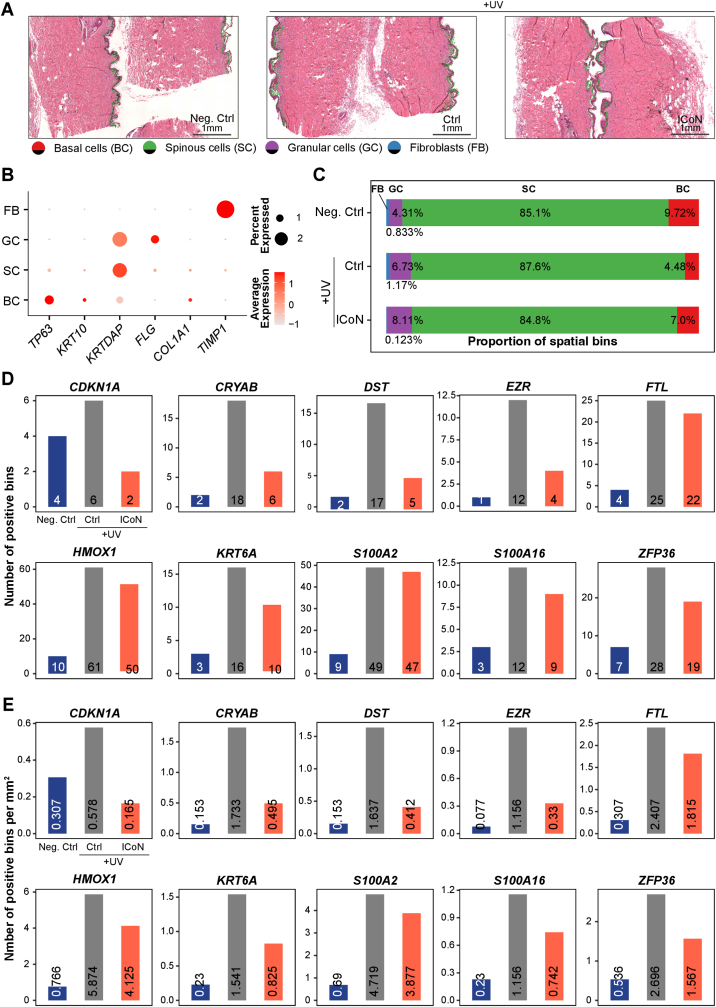
**ICoN Attenuates UV-Induced Histological and Spatial Transcriptomic Features in Aged *Ex Vivo* Human Skin Explant Model**. (**A**) Representative H&E-stained tissue images overlaid with spatial annotation of basal cells (BC), spinous cells (SC), granular cells (GC), and fibroblasts (FB). Scale bar = 1 mm. (**B**) Dot plot of well-established epidermal layer and fibroblast marker genes used to annotate spatial bins, including *TP63* (BC), *KRT10* and *KRTDAP* (SC), *FLG* (GC), and *COL1A1* and *TIMP1* (FB). Dot size indicates the percentage of spatial bins expressing each gene, and color denotes the average scaled expression. (**C)** Stacked bar plots showing the proportion of spatial bins assigned to each cell type across conditions. (**D**) Bar plots of the number of positive spatial bins for senescence- and DNA damage-associated genes, including *CDKN1A*, *CRYAB*, *DST*, *EZR*, *FTL*, *HMOX1*, *KRT6A*, S100A2, *S100A16*, and *ZFP36*. (**E**) The same gene set quantified as the number of positive bins per mm^2^, enabling comparison of spatial expression density across tissues. All samples were derived from a single donor, and two independent tissue sections per condition were analyzed as technical replicates. (For interpretation of the references to color in this figure legend, the reader is referred to the Web version of this article.)

To examine histological and spatial transcriptomic changes associated with ICoN treatment, Visium HD spatial transcriptomic analysis was performed on *ex vivo* aged human skin tissues derived from a single donor, with two independent tissue sections analyzed per condition. Histological examination revealed that UV irradiation induced discernible structural perturbations in the aged epidermis, characterized by increased irregularity of the stratum corneum (Fig. 9A). Conversely, ICoN treatment consistently maintained a more normalized epidermal architecture comparable to the non-irradiated skin tissue, suggesting a protective effect against UV-induced structural remodeling. To establish a molecular baseline, we mapped the cellular landscape using canonical markers such as *TP63* (Basal cells), *KRT10* (Spinous cells), and *FLG* (Granular cells) (Fig. 9B). The spatial distribution of these cell types remained consistent across experimental groups, ensuring that subsequent transcriptomic observations are more likely to reflect altered cellular states rather than shifts in tissue composition (Fig. 9C).

Quantitative profiling of the spatial transcriptome focused on the magnitude of effect and directional consistency across independent tissue sections. UV exposure triggered a substantial and consistent induction of the cellular senescence hallmark *CDKN1A* and the oxidative stress-responsive gene *HMOX1* (Fig. 9D–E). This induction of multiple stress-related transcripts, alongside the upregulation of epidermal activation genes (*KRT6A*) and pro-inflammatory markers (*S100A2*, *S100A16*), defined a coherent transcriptional signature associated with UV-induced damage. Importantly, ICoN treatment consistently attenuated the spatial density and the number of positive bins for these markers across replicates, indicating an attenuation of these transcriptional features.

The spatial feature plots further supported these findings by showing a high degree of spatial plausibility. UV induced over-expression of *DST*, *EZR*, *FTL*, and *ZFP36*, which was diminished in both intensity and distribution following ICoN treatment (Fig. S10). While the small sample size of intrinsic aged human tissue limits formal high-power statistical testing, the observed effect sizes and the spatial alignment of multiple senescence markers are consistent with a potential modulatory effect of ICoN on these transcriptional features.

Together, these observations suggest that liposomal delivery of NAD^+^ is associated with attenuation of UV-associated histological and spatial transcriptomic features in *ex vivo* human skin.

### 2.10 ICoN Reverses Photoaging-associated Transcriptional Dysregulation in *Ex Vivo* Human Skin Explant

To complement the spatial transcriptomic analysis, bulk RNA-seq was performed on UV-irradiated human skin explants to characterize global gene expression patterns associated with UV exposure and ICoN treatment. This approach enabled an overview of gene expression trends, particularly those related to NAD^+^ metabolism, mitochondrial organization, and senescence-associated pathways.

Gene sets were curated based on their previously reported relevance to skin aging and NAD^+^ biology, and their proportional representation was visualized using a pie chart format (Fig. S11A). Bubble and GSEA plots indicated that ICoN treatment was associated with transcriptional enrichment of NAD^+^-related metabolic pathways and attenuation of aging-associated gene signatures, including those linked to mitochondrial dysfunction, inflammatory signaling, and cellular stress responses (Fig. S11B–C, Table S1). These transcriptional trends suggest that ICoN may contribute to modulation of gene expression profiles altered during photoaging. Heatmap analysis further illustrated upregulation of genes associated with reparative and metabolic processes, alongside decreased expression of senescence-associated transcripts across multiple molecular categories (Fig. S11D). While exploratory in nature, these genome-wide signatures point toward a possible shift toward transcriptional normalization in NAD^+^-depleted skin explants following ICoN treatment.

Together, RNA-seq analysis provides descriptive molecular context for the phenotypic alterations observed in human skin explant, supporting the notion that ICoN may modulate age-associated transcriptional programs rather than merely influencing NAD^+^ abundance.

### 2.11 ICoN Extends Lifespan and Improves Healthspan in *C. elegans*

Given the established role of NAD^+^ in longevity, numerous studies have demonstrated that NAD^+^ precursors, such as NR and NMN, can extend lifespan in various model organisms, including *C. elegans*. To investigate whether direct NAD ^+^ delivery via ICoN exerts similar longevity-related effects, we conducted lifespan assays using *C. elegans* exposed to different concentrations of ICoN.

**Fig. 10:**
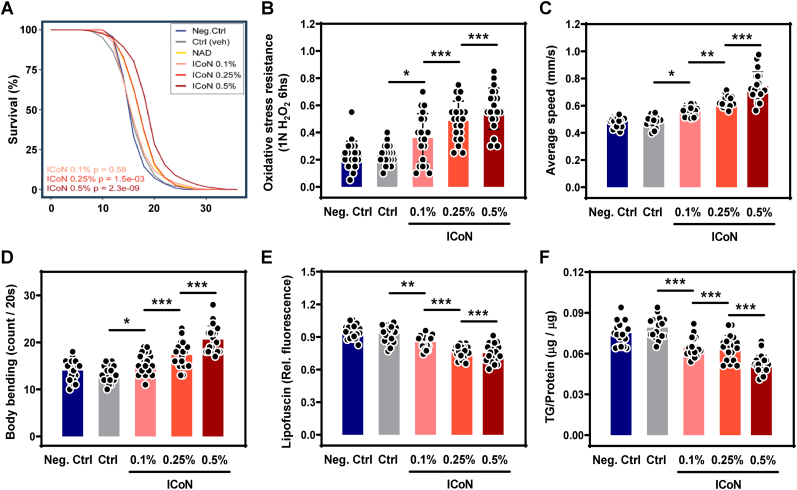
**ICoN Extends Lifespan and Improves Healthspan in *C. elegans***. (**A**) Kaplan-Meier survival curves showing the effect of ICoN on the lifespan of *C. elegans* (n = 20, biological replicates). (**B**) Oxidative stress resistance assay. Wild-type worms in all groups were exposed to 1 N H_2_O_2_, and survival rate was quantified as the percentage of viable animals following oxidative challenge. Increased survival in the ICoN-treated group suggests enhanced oxidative stress resistance, a commonly used functional readout in *C. elegans* aging studies (n = 20, biological replicates). (**C**) Average locomotor speed of *C. elegans* (n = 20, biological replicates). (**D**) Body bending frequency measured over a 20-s interval (n = 20, biological replicates). (**E**) Quantification of lipofuscin accumulation assessed by autofluorescence intensity (n = 20, biological replicates). (**F**) Triglyceride-to-protein (TG/protein) ratio in *C. elegans* (n = 20, biological replicates).

Lifespan analysis was conducted across multiple concentrations of ICoN to assess dose-dependent effects. Survival curves revealed that ICoN supplementation resulted in a significant extension of lifespan in a dose-dependent manner, with the most pronounced effects observed at 0.25% and 0.5% concentrations (Fig. 10A). However, a lower concentration of 0.1% did not produce a significant effect, indicating a threshold for efficacy.

To assess whether lifespan extension by ICoN was accompanied by functional healthspan benefits, a comprehensive panel of physiological and stress-resilience parameters was evaluated in *C. elegans*. Oxidative stress tolerance was significantly enhanced in a dose-dependent manner, as shown by improved survival following 1 N H_2_O_2_ exposure (Fig. 10B). Locomotor performance, measured through average speed and body bending frequency, were improved by ICoN treatment (Fig. 10C–D), suggesting preservation of functional capacity under stress conditions. Moreover, lipofuscin accumulation and triglyceride-to-protein ratios, indicators of oxidative burden, were reduced in ICoN-treated worms (Fig. 10E–F). Consistently, heat stress survival (35 °C for 16 h) was significantly increased by ICoN, further demonstrating enhanced stress tolerance (Fig. S12).

Together, these findings demonstrate that ICoN is associated with improved lifespan- and healthspan-related phenotypes in *C*. *elegans* under both physiological and stress states.

## 3 Discussion

Achieving durable rejuvenation of aging skin requires more than simply raising systemic NAD^+^ levels; it demands precise restoration of NAD^+^-dependent homeostasis within the dermal niche. In this study, we show that a rationally designed, ionically stabilized transfersome, ICoN, can locally replenish NAD^+^ in skin, recalibrating mitochondrial function, redox balance, and regenerative capacity in dermal fibroblasts and attenuating UV-induced senescence in human skin explants. The convergence of cellular, tissue, organismal, and transcriptomic data supports a model in which spatially targeted NAD^+^ delivery can re-engage conserved pro-longevity and pro-regenerative programs that are otherwise inaccessible to conventional formulations.

Importantly, recent evidence suggests that NAD^+^ depletion in cutaneous tissue is not merely systemic but occurs locally within dermal fibroblasts, where age-related declines in NAD^+^ and NADPH have been directly quantified in human skin biopsies. Despite not being classically defined as highly energy-demanding cells, fibroblasts undergo metabolic and mitochondrial decline during aging, exhibiting reduced respiratory capacity, impaired OXPHOS gene expression, and diminished bioenergetic output, features that can be partially restored through NAD^+^ augmentation [40]. Senescent fibroblasts further display elevated oxidative stress, disrupted autophagy, and sustained mTORC1 activity, which are all tightly linked to NAD^+^/redox imbalance and closely associated with ECM instability [41]. Additionally, decreased activity of NAD^+^ dependent sirtuins, such as SIRT1 and SIRT6, has been implicated in collagen loss, inflammatory remodeling, and impaired regenerative capacity of fibroblasts within aged skin [42]. These studies collectively position dermal fibroblasts as metabolically vulnerable cells in aging skin and reinforce the relevance of NAD^+^ repletion as a strategy for preserving their structural and functional competence. In this regard, although fibroblasts are not initially metabolically demanding, aging progressively shifts them into a metabolic bottleneck that governs ECM turnover, redox balance, and inflammatory remodeling, thereby positioning NAD^+^ availability as a rate-limiting determinant of dermal homeostasis.

A key implication of our work is that the limited impact of systemic NAD^+^ supplementation on skin is not solely a question of dosing but of pharmacokinetic inaccessibility. The skin combines multiple layers of resistance to systemically delivered, hydrophilic, and charged metabolites such as NAD^+^ [43]. The stratum corneum forms a tightly ordered lipid-protein lamella that restricts diffusion of charged molecules even when circulating levels are elevated. Beneath this barrier, cutaneous blood flow is tightly autoregulated and comparatively low, limiting the fraction of systemically administered agents that can equilibrate within dermal tissue. The dermal extracellular matrix, enriched in crosslinked collagen and hyaluronan, further acts as a molecular sieve that sequesters or slows diffusion of macromolecules before they reach fibroblasts [44,45]. Together, these features render the dermis a pharmacokinetic sink rather than a passive recipient of systemic exposure, explaining why even potent systemic NAD^+^ boosters show modest and variable effects on skin structure and function. In this context, the value of ICoN lies not only in stabilizing a labile metabolite, but in providing a delivery architecture that is intrinsically matched to the anatomical and microenvironmental constraints of the dermis.

The design of ICoN also has broader implications for how labile, highly polar metabolites can be pharmacologically harnessed. By combining electrostatic complexation of NAD^+^ with deformable lipid vesicles, ICoN enhances both transdermal passage and intracellular availability, enabling sustained engagement of NAD^+^-dependent pathways without relying on prodrugs or intracellular biosynthesis alone. This conceptual framework may be generalizable to other charged metabolites or cofactors that have been difficult to exploit therapeutically because of poor membrane permeability and rapid degradation.

Moreover, the *C. elegans* assays provide an additional organismal context in which the phenotypic effects of ICoN can be examined. Although this model does not recapitulate mammalian skin physiology or transdermal delivery, the observed lifespan extension and improved stress resilience suggest that ICoN is associated with broader biological effects beyond a single tissue setting. These findings are not intended as direct translational evidence, but they indicate that the impact of ICoN is not restricted to localized cellular responses.

Despite these promising effects, several limitations must be addressed for clinical translation. This study primarily relies on *in vitro* and skin explant models, which, while informative, do not fully capture systemic NAD^+^ metabolism. Although the FK866-induced acute NAD^+^ depletion model provides a controlled framework to interrogate NAD^+^-dependent regulatory pathways, it does not fully reflect the cumulative and multifactorial nature of physiological skin aging. In addition, the spatial transcriptomic analysis was performed using tissue sections derived from a single donor, lacking biological replication. As a result, these data should be interpreted as exploratory, with conclusions drawn primarily from consistent spatial patterns and effect sizes rather than formal statistical inference. Therefore, further investigations using chronological aging models or long-term artificial aging models are warranted to validate the sustained efficacy and clinical translatability of ICoN in a more comprehensive physiological context. Future studies should incorporate animal models to evaluate the pharmacokinetics, biodistribution, and long-term effects of ICoN under physiological conditions. In addition, the formulation's stability, retention in target tissue, and potential immunogenicity upon repeated application require further assessment. Optimizing ICoN for controlled release and sustained NAD^+^ availability could further improve its therapeutic efficacy.

A critical next step involves conducting human clinical trials to assess the safety, efficacy, and durability of ICoN in improving skin health, metabolic function, and aging-related phenotypes. Evaluating its utility in populations with metabolic disorders or accelerated aging syndromes will help delineate its clinical relevance. Collectively, these findings establish ICoN as a next-generation NAD^+^ delivery platform with broad therapeutic potential. By enhancing NAD^+^ bioavailability, restoring cellular homeostasis, and mitigating oxidative stress and senescence, ICoN presents a compelling strategy for managing aging-related diseases and promoting healthy skin aging. The observed lifespan extension and healthspan enhancement in *C. elegans* further support its application in age-associated disorders.

## 4 Materials and methods

### 4.1 Reagents and materials

All reagents and materials used in this study were of analytical grade and sourced from reputable suppliers. Dipropylene glycol (DPG) was obtained from SamKwang-Chem Corp. (Yongin-si, Gyeonggi-do, Republic of Korea). Hydrogenated lecithin was purchased from Neuropid Co., Ltd. (Uiwang-si, Gyeonggi-do, Republic of Korea), while cholesterol was supplied by Active Concepts LLC (Lincolnton, NC, USA). Sorbitan oleate was provided by Croda Inc. (East Yorkshire, UK). Sodium stearoyl glutamate (SSG) was sourced from Ajinomoto Co., Inc. (Tokyo, Japan). Nicotinamide adenine dinucleotide (NAD^+^) was obtained from GFC Life Science (Hwaseong-si, Gyeonggi-do, Republic of Korea). 1,2-Hexanediol was acquired from Osaka Organic Chemical Ind., Ltd. (Tokyo, Japan). Cell culture reagents, including Dulbecco's Modified Eagle Medium (DMEM), phosphate-buffered saline (PBS, pH 7.4), fetal bovine serum (FBS), penicillin-streptomycin (P/S), sodium pyruvate, and L-glutamine, were obtained from Gibco (Waltham, MA, USA). Calcium chloride (CaCl_2_) was supplied by Sigma-Aldrich Co. (St. Louis, MO, USA). Trypsin-ethylenediaminetetraacetic acid (EDTA) was purchased from Thermo Fisher Scientific Inc. (Waltham, MA, USA). All chemicals and reagents were stored under manufacturer-recommended conditions to ensure stability and functionality.

### 4.2 Preparation and characterization of ICoN

ICoN was formulated following the composition outlined in Table S2 and Fig. S13. The preparation process involved a stepwise assembly of the lipid-based vesicle system to ensure optimal encapsulation efficiency and stability. Phase A lipids were completely dissolved at 80 °C to achieve homogeneity. Once fully solubilized, Phase B was introduced into Phase A, followed by continuous stirring for 30 min at 80 °C to facilitate transfersome formation. Subsequently, Phase C was introduced into the transfersome suspension and stirred for an additional 10 min, after which the temperature was gradually reduced to room temperature to stabilize the formulation.

Phase D, containing various concentrations of NAD^+^, was separately prepared by maintaining a mixing temperature of 30-40 °C. Finally, ICoN was assembled by carefully combining the pre-formed transfersomes with the NAD^+^ mixture (Phase D), leading to the formation of the final ICoN complex. This optimized process ensured efficient encapsulation of NAD^+^ within the transfersome vesicles, enhancing both its stability and therapeutic bioavailability.

The physicochemical properties of ICoN were evaluated to determine its stability and functional efficacy. The mean particle diameter and zeta potential of the ICoN complexes were measured using a Zetasizer Nano-ZS90 (Malvern Instruments Ltd., Malvern, UK) employing the dynamic light scattering (DLS) method. In addition, NTA analysis was performed using a NanoSight Pro (Malvern Panalytical, Grovewood Road, Malvern, UK).

The loading efficiency of NAD^+^ into ICoN was determined by separating free NAD^+^ from ICoN using a centrifugal filter (10K MWCO, Hangzhou Cobetter Filtration Equipment Co., Ltd., Hangzhou, Zhejiang, China). Briefly, 5 mL of ICoN solution was loaded into a centrifugal filter tube and centrifuged at 4000×*g* for 30 min at 4 °C in a swing-type rotor. The filtrate containing unloaded NAD^+^ was collected and quantified by HPLC. HPLC analysis was performed at a flow rate of 1 mL min^−1^ with an injection volume of 10 μL, using a gradient of 0.5 M phosphate buffer (solvent A) and methanol B (solvent B), as previously reported [46]. Loading efficiency (%) was calculated as [(total weight of NAD^+^ – the weight of free NAD^+^ in filtrate)/total weight of NAD^+^] × 100.

FRET analysis was conducted as previously reported [47]. Briefly, FRET pair dyes (FSD 488 and FSD 555) were conjugated to NAD^+^ and blank liposomes, respectively, using a fluorescent labeling kit (BioActs, Incheon, Republic of Korea). Fluorescence emission spectra were recorded using a microplate reader (Varioskan™ LUX Multimode Microplate Reader, Thermo Fisher Scientific, USA), and FRET efficiency was calculated based on changes in donor and acceptor fluorescence intensities. FTIR spectroscopy was conducted using a Spectrum Two FTIR spectrometer (PerkinElmer, USA). Samples including NAD^+^, blank liposomes, ICoN, hydrogenated lecithin, and sodium stearoyl glutamate (SSG) were prepared under identical conditions. The region between 1700 and 1400 cm^−1^ was examined to assess changes in carboxyl group–related vibrational features [48].

### 4.3 Morphological analysis of ICoN using field emission scanning electron microscopy (FE-SEM)

The structural and morphological properties of ICoN were examined using FE-SEM (S-4700, Hitachi, Japan), following previously established protocols. Prior to imaging, samples were carefully prepared to preserve the integrity of the nanostructures, ensuring high-resolution visual representation. The analysis was conducted under a high-vacuum mode to prevent sample contamination and enhance image clarity. Imaging parameters were set to an acceleration voltage of 20 kV and a working distance of 10 mm, with a magnification of 100,000 × , allowing for detailed observation of ICoN's surface topography and nanoparticle distribution. This high-resolution imaging approach provided critical insights into the physical morphology, homogeneity, and stability of ICoN, validating its structural integrity for further applications.

### 4.4 Transdermal delivery assessment of ICoN

To assess the transdermal delivery efficiency of ICoN, the Franz diffusion cell method was employed using porcine skin (conventional grade micropig, 6-8 months old, back region) obtained from APURES Co., Ltd. (Pyeongtaek-si, Gyeonggi-do, Republic of Korea). The excised skin was prepared to a thickness of approximately 1000 μm prior to mounting. Briefly, the skin was mounted between the donor and receptor compartments of a Franz diffusion cell, with the stratum corneum facing the donor side. The effective diffusion area was approximately 3.14 cm^2^ (2.0 cm diameter). The receptor chamber (2.7 mL) was filled with PBS containing 0.1% methyl paraben and maintained at 37 °C under continuous stirring (300 rpm). Either ICoN or free NAD^+^ was applied to the donor compartment, which was sealed to maintain hydration. Transdermal penetration was evaluated after 24 h of incubation. All porcine skin explants were trimmed to matched baseline characteristics prior to analysis to enable direct comparison across groups.

To quantify NAD^+^ penetration, samples collected from the receptor chamber were diluted (1:10) with a 1:1 water/methanol mixture to facilitate NAD^+^ extraction. Unabsorbed NAD^+^ from the skin surface was thoroughly removed, while skin-absorbed NAD^+^ was extracted by homogenizing the excised skin tissues in a 1:1 water/methanol solution using a Precellys 24 homogenizer (Bertin Technologies, Montigny-le-Bretonneux, France).

Additionally, transdermal penetration studies were conducted using ICoN-incorporated cosmetic formulations. Fluorescently labeled NAD^+^ or ICoN was incorporated into an essence cream at a final NAD^+^ concentration of 0.01% and applied to the skin. In all transdermal delivery experiments involving NAD^+^ and ICoN, fluorescently labeled NAD^+^ was used. Fluorescent labeling of NAD^+^ was performed using the FSD Fluor™ 488 NHS Ester Labeling Kit. The fluorescence intensity of transmitted NAD^+^ was measured with an excitation wavelength of 530 nm and an emission range of 550–700 nm using a Varioskan LUX Multimode Microplate Reader (Thermo Fisher Scientific Inc., Waltham, MA, USA).

To visualize and quantify transdermal NAD^+^ penetration, an Ultrafast IntraVital Confocal Microscope (IVM-CMS3, IVIM Technology, Inc., Daejeon, Republic of Korea) was employed. Fluorescence signals were analyzed in both 2D and 3D formats using Studio Visual Software (IVIM Technology, Inc., Daejeon, Republic of Korea), providing a high-resolution spatial distribution profile of NAD^+^ delivery facilitated by ICoN within skin layers.

### 4.5 Cell culture and cellular senescence assay

Human skin fibroblast cells (HS68) were obtained from the American Type Culture Collection (ATCC, Manassas, VA, USA). Cells were maintained in DMEM supplemented with 10% FBS, 1% P/S, 1 mM sodium pyruvate, and 2 mM L-glutamine at 37 °C in a humidified atmosphere containing 5% CO_2_. To optimize culture conditions, CaCl_2_ was added to the medium at final concentrations of 1.8 mM. HS68 cell viability or relative cell population was measured by the CCK-8 assay according to the manufacturer's protocol (Cell Counting Kit-8, Dojindo, Kumamoto, Japan).

Cellular senescence was assessed by measuring β-galactosidase activity, a widely used biomarker in previous studies. For quantitative analysis, a plate-based β-galactosidase quantification assay was performed using the CellEvent™ Senescence Green Detection Kit (C10850, Thermo Scientific, Waltham, MA, USA). For fluorescence analysis, cells were examined using a CytoFLEX flow cytometer (Beckman Coulter, Brea, CA, USA). The mean fluorescence intensity per cell and the percentage of β-galactosidase-positive cells were quantified to determine the degree of cellular senescence. A total of 10,000 cells were analyzed.

### 4.6 Quantification of NAD^+^ and ATP

The PicoSens NAD/NADH Assay Kit (BM-NDH-100, Biomax, Guri-si, Republic of Korea) was used to quantify cellular NAD^+^ according to the manufacturer's protocol. Following a 24-h application of test materials to fibroblasts, the cell culture supernatant was removed. Cells were washed three times with PBS and lysed using the provided lysis buffer. The lysed cell samples were centrifuged, and the resulting supernatants were divided into two microtubes: one for measuring total NAD^+^ (NAD^+^+ NADH) and the other for measuring NADH. To quantify NADH, the designated microtubes were heated at 60 °C for 30 min. Enzymatic reactions were then performed according to the manufacturer's instructions to determine total NAD^+^ and NADH levels. The amount of NAD^+^ was calculated by subtracting the NADH value from the total NAD^+^. For quantification of NAD^+^ in skin explants, tissues were homogenized with Precellys 24 homogenizer (Bertin Technologies, Montigny-le-Bretonneux, France), and centrifuged. The clear supernatants were recovered and analyzed.

For ATP quantification, the PicoSens ATP Assay Kit (BM-ATP-100, Biomax, Guri-si, Republic of Korea) was used. Following a similar protocol to NAD^+^ measurement, cells were lysed, and ATP levels were determined via enzymatic reaction.

### 4.7 Quantitative analysis of aging-related gene expression

To evaluate the expression levels of aging-related genes, quantitative reverse transcription polymerase chain reaction (qRT-PCR) was conducted. Total RNA was extracted from human skin fibroblast cells using the AccuPrep® Universal RNA Extraction Kit (BIONEER Co., Daejeon, Republic of Korea), following the manufacturer's protocol. Subsequently, 1 μg of extracted RNA was reverse-transcribed into complementary DNA (cDNA) using the TOPscript™ cDNA Synthesis Kit (dN6 Mix) (Enzynomics Co., Ltd., Daejeon, Republic of Korea), in accordance with the manufacturer's instructions. Reverse transcription reactions were carried out using a Veriti™ 96-Well Thermal Cycler (Applied Biosystems, Waltham, MA, USA). qRT-PCR was then performed using the StepOnePlus™ Real-Time PCR System (Applied Biosystems, Waltham, MA, USA) and the AccuPrep® GreenStar™ qRT-PCR PreMix Kit (BIONEER Co., Daejeon, Republic of Korea), following the manufacturer's guidelines. The thermocycling conditions for qRT-PCR were as follows: 30 cycles at 95 °C for 45 s, 60 °C for 1 min, and 72 °C for 45 s.

### 4.8 Assessment of sirtuin activation

Sirtuin activation was analyzed using a commercially available assay kit with slight modifications (SIRT1 (Sirtuin1) Fluorogenic Assay Kit, 50081, BPS Bioscience, San Diego, CA, USA). A master mix was prepared according to the manufacturer's protocol, consisting of 5 μL of HDAC solution, 0.01 μL of NAD^+^, 5 μL of BSA (1 mg/mL), 19.5 μL of SIRT assay buffer, and 100 μM HDAB substrate. Cell lysates were obtained using lysis buffer (M-PE™ Mammalian Protein Extraction Reagent, 78501, Thermo Scientific, CA, USA). Total protein concentration was quantified using the BCA assay (Protein Quantification – BCA, BCA0500, Biomax, South Korea). Cell lysates were diluted to equal protein concentrations across samples before further analysis. For the assay, 20 μL of each cell lysate sample was mixed with the master mix and incubated at 37 °C for 30 min. Following incubation, 50 μL of SIRT assay developer was added, and the plate was incubated at room temperature for 15 min. Fluorescence intensity was measured at an excitation/emission wavelength of 360/450 nm using a microplate spectrophotometer.

### 4.9 DNA damage analysis using comet assay and γH2A.X assay

Single cell DNA strand breaks were estimated with comet assay. It was performed by a commercially purchased kit from Abcam (ab238544, Cambridge, UK). The Hs68 cells were seeded on 6-well cell culture plate at a density of 2 × 10^5^ cells/well in 2 mL of culture medium for 24 h. Prior to UV irradiation, the medium was exchanged, and the cells were treated with compounds of interest (NAD^+^, ICoN) for 24 h. Then, the cells were irradiated with UVB (40 mJ) and re-treated for 24 h. Cells were collected and suspended with DPBS/Agarose mix. The agarose/cell mixtures were set onto comet slides, and treated with lysis buffer (45 min) and alkaline electrophoresis solution (30 min) following the manufacturer's protocol. Electrophoresis was performed under alkaline condition with 1V/cm for 15 min. After electrophoresis, the slides were washed with water and pre-chilled 70% Ethanol. After drying, the slide was stained with Vista Green DNA Dye for 15 min. The fluorescence images were obtained with fluorescence microscopy using a FITC filter. The Tail DNA intensity and the tail moment length, which reflect DNA damages of host cells were analyzed by CaspLab software (1.2.3b1). Results were presented as Tail DNA (%) and Olive Tail Moment by the following formula.

Tail DNA (%) = Tail DNA intensity / Cell DNA intensity × 100

Olive Tail Moment = Tail DNA (%) / Tail moment length

The γH2A.X assay was used to evaluate phosphorylation of histone H2A.X at serine 139 following DNA damage. It was performed by a commercially purchased kit from Abcam (ab242296, Cambridge, UK). The Hs68 cells were seeded on 12-well cell culture plate at a density of 5 × 10^4^ cells/well in 1 mL of culture medium for 24 h. Prior to UV irradiation, the medium was exchanged, and the cells were treated with compound of interest (NAD^+^, ICoN). After 24 h of incubation, the cells were irradiated with UVB (40 mJ) and re-treated for 24 h. Then, the medium was aspirated off and the cells were washed with DPBS (DPB001, Solbio, Seoul, Republic of Korea). 4% paraformaldehyde (P2031, Biosesang Inc., Gyeonggi-do, Republic of Korea)/DPBS (DPB001, Solbio, Seoul, Republic of Korea) was used to fix the cells. After fixation, ice-cold 90% methanol (1.06009.1011, Merck, Darmstadt, Germany) and blocking solution was added in sequence. *Anti*-phospho-histone H2A.X antibody solution was treated to each well for 1 h at a room temperature. The cells were washed with PBST, and treated with secondary antibody, FITC conjugate solution for 1 h at a room temperature. The fluorescence images were obtained with fluorescence microscopy using a FITC filter after sufficient washing.

### 4.10 Assessment of cellular oxidative stress using DCFDA assay

Cellular oxidative stress was assessed using the DCFDA assay, performed with a commercially available kit (ab113851, Abcam, Cambridge, UK). Hs68 cells were seeded in a black, flat, clear-bottom 96-well plate at a density of 2 × 10^4^ cells/well in 100 μL of culture medium and incubated for 24 h. After replacing the medium with fresh culture medium, cells were treated with the compound of interest (NAD^+^ or ICoN) for an additional 24 h. Subsequently, cells were incubated with DCFDA solution (20 μM) in fresh medium for 3 h to allow ROS staining. The medium was then aspirated, and H_2_O_2_ solution (500 μM, 4158-4400, DAEJUNG, Gyeonggi-do, Republic of Korea) containing the compound of interest was added. After a 45-min incubation, fluorescence intensity was measured at an excitation/emission wavelength of 485/535 nm in the presence of the compound or control medium.

### 4.11 Mitochondrial respiration assay

Mitochondrial respiration was assessed using the Seahorse XFe96 Analyzer (Agilent Technologies) with the XF Cell Mito Stress Test kit. Cells were seeded at 2 × 10^4^ cells per well and allowed to reach optimal confluency prior to the assay. Prior to measurement, culture medium was replaced with Seahorse XF assay medium (supplemented with 1 mM pyruvate, 2 mM glutamine, and 10 mM glucose) and incubated for 30 min in a CO_2_-free environment. Sequential injections were performed as follows: oligomycin (1 μM), FCCP (1.5 μM), and rotenone/antimycin A (0.5 μM each). To ensure experimental consistency and to avoid bias from potential differences in proliferation or cell size, OCR values were normalized to 1 × 10^4^ cells per well. All procedures were conducted strictly following the manufacturer's standardized operational guidelines to ensure reproducibility and technical robustness.

### 4.12 Label-free 3D live imaging analysis of mitochondria

Hs27 human foreskin fibroblasts (ATCC, #7006836) were maintained in DMEM supplemented with 10% fetal bovine serum under standard culture conditions (37 °C, 5% CO_2_, humidified atmosphere). HS27 fibroblasts were selected exclusively for Tomocube-based live-cell senescence imaging and OCR analysis, as they show reproducible mitochondrial elongation upon senescence induction and maintain optical viability under real-time imaging conditions. Cellular senescence was induced by repeated exposure to 150 nM H_2_O_2_ for 2 h per day over 4 consecutive days, a protocol designed to mimic oxidative stress-associated senescence.

To assess mitochondrial structural changes induced by ICoN treatment, we used label-free live-cell 3D holotomography (HT-X1; Tomocube Inc., Daejeon, Korea), an optical diffraction tomography (ODT)-based system that acquires multiple holographic projections under varying illumination angles and reconstructs a three-dimensional refractive index (RI) tomogram of each cell by solving the inverse light-scattering equation. This allows high-resolution visualization of subcellular organelles without fluorescent labels and has been validated for mitochondrial imaging in multiple studies [49,50].

Cells were treated with either vehicle-Transfersome or ICoN (1%) and immediately subjected to real-time 3D holotomography at 37 °C in a 5% CO_2_ incubated environment. Images were acquired at 30-s intervals to continuously monitor dynamic changes in mitochondrial structure. Live-cell imaging was conducted for 30 min in normal cells, whereas senescent cells were monitored for 60 min to compensate for their diminished physiological activity and slower morphological transitions.

Mitochondria were segmented using TomoAnalysis software (Tomocube Inc.), which applies RI-based thresholding combined with AI-driven morphological feature extraction to distinguish mitochondria from other organelles based on: (i) Refractive index range specific to mitochondrial membranes; (ii) Tubular or reticular morphology; (iii) Spatial continuity within cytosolic compartment. The segmentation pipeline follows the manufacturer's validated algorithm for mitochondrial identification, and similar approaches have been reported for label-free mitochondrial quantification. Each segmented mitochondrial unit was computationally analyzed to extract structural parameters, including skeleton length, surface area, volume, and total mitochondrial count per cell. These metrics were used to classify mitochondria into fragmented, intermediate, and elongated subpopulations based on morphological signatures previously established in mitochondrial dynamics research. Endpoint structural data were obtained 1.5 h post-treatment.

All segmentation parameters and RI thresholds were applied uniformly to all experimental groups to ensure reproducibility. The biological interpretation of structural changes was cautiously made in combination with independent bioenergetic assays (OCR, ATP content) to distinguish between fragmentation linked to dysfunction versus transitional reorganization.

### 4.13 Flow cytometric and spectrophotometric analysis of mitochondrial function

Mitochondrial membrane potential and superoxide levels were assessed using TMRM (Tetramethylrhodamine, methyl ester) and MitoSOX™ assays, respectively. HS68 cells were seeded into six-well plates at a density of 3 × 10^5^ cells/well and incubated at 37 °C for 24 h. The cells were then treated with FK866 for 24 h to induce NAD^+^ depletion, followed by treatment with NAD^+^ or ICoN for an additional 48 h.

For mitochondrial membrane potential analysis, cells were seeded into black-walled 96-well plates. To establish a baseline control, FCCP (20 μM, C2920, Sigma-Aldrich, St. Louis, MO, USA) was added to serum-free media 15 min before staining. Cells were then stained with 20 μM TMRM (T668, Invitrogen, Carlsbad, CA, USA) in HBSS for 45 min, followed by two washes with HBSS. Nuclei were counterstained with Hoechst 33342 (H3570, Thermo Scientific, Waltham, MA, USA). Fluorescence intensities were measured using a spectrophotometer in well-reading mode at Ex/Em = 548/573 nm for TMRM and Ex/Em = 352/454 nm for Hoechst 33342. Two complementary approaches were used for quantitative analysis of ΔΨm, following established protocols for TMRM-based mitochondrial polarization assays: (1) End-point ΔΨm assessment (Fig. 5B): ΔΨm was calculated using the FCCP-treated group as a reference baseline according to the equation: $\Delta \Psi_{m}=\left(F_{\text{treatment}}-F_{\text{FCCP}}\right)$. (2) Real-time TMRM kinetics (Fig. 5C): To monitor dynamic mitochondrial responses, TMRM fluorescence was recorded every 0.1 s immediately after FCCP addition using kinetic reading mode. These values represent absolute fluorescence intensity (A.U.) rather than normalized ΔΨm. Time-resolved decay profiles were analyzed to assess membrane stability and response to ICoN-mediated NAD^+^ supplementation.

For mitochondrial superoxide detection, HS68 cells were washed with DPBS and incubated with 5 μM MitoSOX™ Mitochondrial Superoxide Indicator (M36008, Invitrogen, Carlsbad, CA, USA) for 30 min in the dark. Cells were then washed with DPBS, detached using Accutase Cell Detachment Solution (A6964, Sigma-Aldrich, St. Louis, MO, USA), and washed twice. The collected cells were analyzed using a CytoFLEX Flow Cytometer (Beckman Coulter, Brea, CA, USA), and the mean fluorescence intensity was quantified to assess mitochondrial superoxide levels. A total of 10,000 cells were analyzed.

### 4.14 Cell migration and cell proliferation assay

For all functional assays, cells were seeded at identical densities after viability adjustment using trypan blue exclusion to normalize for potential differences in proliferative rate across groups. This ensured equal baseline cell numbers regardless of treatment history. Proliferation curves were established by counting viable cells at predefined timepoints using a hemocytometer and automated live/dead discrimination. Data were fitted using exponential growth models to compare replication dynamics between groups.

Fibroblasts were plated in 24-well plates. After one day, 10 μM FK866 (Daporinad, a noncompetitive inhibitor of nicotinamide phosphoribosyl transferase (NAMPT), CAS 658084-64-1) was added, and the cells were incubated for an additional 15 h. Following this, the culture supernatants were removed, and uniform scratches were created using a scratcher (SPLScar™ Scratcher, SPL, Pocheon, Republic of Korea). The cells were then washed once with PBS and replenished with culture media containing the test substances. Wound areas were evaluated under a microscope after 15 h.

Cell proliferation was measured by CCK-8 as previously described. Replicative lifespan was mathematically calculated. In the nonlinear regression logistic growth model, the time at which cells reach 95% of saturation was calculated.

### 4.15 Nitrogen oxide (NO) production and β-hexosaminidase activity assay

RAW 264.7 cells were seeded in 24-well plates and incubated for 12 h. After incubation, the media in each well were aspirated and replaced with fresh FBS-free DMEM. Various concentrations of ICoN and vehicle were prepared in FBS-free DMEM to a final volume of 500 μL per well. Cells were further incubated for 24 h. As a positive control, 1 μg/mL lipopolysaccharide (LPS) were treated for 24 h. To assess nitric oxide production, 100 μL of culture supernatant was transferred to a 96-well plate, followed by the addition of an equal volume of Griess reagent (0.04 g/ml, Sigma-Aldrich Co., Saint Louis, MO, USA). The plate was then incubated in the dark at room temperature for 15 min. Absorbance was measured at 540 nm using microplate reader. Nitrite concentrations were calculated based on a sodium nitrite (NaNO_2_) standard curve.

The inhibitory activity of the test agents on β-hexosaminidase release from RBL-2H3 cells was evaluated. RBL-2H3 cells were seeded in 24-well plates and incubated overnight at 37 °C with 5% CO_2_. After washing twice with serum-free medium, the cells were incubated for 2 h with the test agents with and without 60 μg/mL compound 48/80 (C48/80) at the indicated concentrations in serum-free medium. The reaction was terminated by placing the plates on ice for 10 min. The 24-well plates were centrifuged at 200×*g* for 5 min at 4 °C, and 50 μL aliquots of the supernatant were transferred to 96-well plates. To determine total β -hexosaminidase content, supernatants were mixed with an equal volume of substrate solution containing p-nitrophenyl-N-acetyl-β-D-glucosaminide in 0.1 M citric acid/sodium citrate buffer (pH 4.5) and incubated for 90 min at 37 °C. The enzymatic reaction was stopped by adding stop buffer (0.1 M sodium carbonate/sodium bicarbonate, pH 10.5). β-hexosaminidase release was quantified by measuring absorbance at 405 nm using a microplate spectrophotometer.

### 4.16 RNA-sequencing and Gene Set Enrichment Analysis (GSEA)

RNA-sequencing and GSEA were conducted to identify differentially expressed genes and enriched biological pathways as described [51]. RNA-seq analysis, including RNA isolation, library preparation, and sequencing, was performed by Theragenbio, Inc. Data quality was assessed using FastQC, and sequencing reads were aligned to the human reference genome (GRCh38) using STAR aligner. Gene expression levels were quantified in Transcripts Per Million (TPM), and log_2_-transformed TPM values were used for downstream analysis.

GSEA was conducted using the Broad Institute's Gene Set Enrichment Analysis software (www.gsea-msigdb.org) to evaluate pathway-level changes. Enrichment scores, p-values, and false discovery rate (FDR) q-values were computed to assess statistical significance. Data visualization, including enrichment plots and heatmaps, was performed in RStudio using *ggplot2* and *pheatmap* packages (www.r-project.org). Statistical analyses, including Student's *t*-test and Levene's test, were applied to assess gene expression differences between experimental groups.

### 4.17 Immunocytochemistry and immunohistochemistry assay

Cells were fixed with 4% paraformaldehyde (P2031, Biosesang Inc., Gyeonggi-do, Republic of Korea) in DPBS. To permeabilize the cells, 0.5% Triton X-100 (T9284, Sigma-Aldrich, St. Louis, MO, USA) in DPBS (DPB001, Solbio, Seoul, Republic of Korea) was applied for 20 min at room temperature. After washing with DPBS, cells were sequentially incubated with a primary antibody (*Anti*-Ki67, ab15580, Abcam, Cambridge, UK) and a secondary goat anti-rabbit IgG antibody conjugated with Alexa Fluor 488 (ab150077, Abcam, Cambridge, UK). DNA was counterstained with DAPI (D9542, Sigma-Aldrich, St. Louis, MO, USA). Fluorescent signals were analyzed using a CytoFLEX flow cytometer (Beckman Coulter, CA, USA), and the mean fluorescence intensity per cell was quantified. A total of 10,000 cells were analyzed.

Full-thickness human skin samples, excluding adipose tissue, were obtained from Biopredic (Saint-Grégoire, France) through a legally and ethically approved process. The skin samples (Cat. DISC1D10, Batch DISC1D10L150, Sample No. PEA09724460732) had the following donor information: Sex (Female), Age (37, 60), Biopsy Site (Abdomen), HIV1/2, HBV, and HCV status (undetectable), and no other donor restrictions. Samples were shipped in gauze soaked with transport medium at 4 °C on the day of or the day following surgery. Test materials were topically applied to the skin biopsies every three days, with culture media (Cat. MIL215C) refreshed at the same interval. To induce skin aging conditions, ultraviolet (UVA: 100 mJ/cm^2^, UVB: 70 mJ/cm^2^) irradiation was applied every three days using a BIO-SUN irradiation system (Vilber Lourmat, Marne-la-Vallée, France). Skin biopsies were incubated at 37 °C in 5% CO_2_. After 14 days of treatment, skin tissues were fixed with 4% paraformaldehyde (P2031, Biosesang Inc.), embedded in paraffin blocks, and stained using Masson's trichrome staining. For immunohistochemistry analysis of p16, p21 and Ki67 expression in skin tissues, primary antibodies (p16: ab108349, p21: ab220206, Ki67: ab15580 Abcam, Cambridge, UK) were used. Images of stained human skin sections were captured using the EVOS™ FL Auto2 Imaging System (Thermo Fisher Scientific, Waltham, MA, USA). Skin biopsies were stained using Masson's trichrome to visualize cells and collagen within the tissue for investigation of epidermis enhancement.

### 4.18 Visium HD spatial transcriptomics analysis

Human skin tissue samples were analyzed using Visium HD spatial transcriptomics (10x Genomics). A total of six samples were included, comprising three experimental conditions with two independent samples per condition: negative control (Neg. ctrl), UV-damaged control (ctrl), and UV-damaged with ICoN treatment (ICoN). All samples were derived from human skin tissue obtained from a single donor (Abdomen, 65 years-old, Female, Caucasian). Independent tissue sections were processed and analyzed separately and were treated as technical replicates at the tissue-section level rather than biological replicates.

Spatial transcriptomic profiling was performed using the 10x Genomics Visium HD Spatial Gene Expression platform, a probe-based spatial transcriptomics technology enabling high-resolution, whole-transcriptome RNA profiling at 8 μm square bin resolution. Libraries were prepared according to the manufacturer's protocol and sequenced on an Illumina MiSeq 2000 system using paired-end sequencing. Raw sequencing data were processed with Space Ranger (v3.1.3, 10x Genomics). Reads were aligned to the human reference genome (GRCh38-2020-A) using the Visium Human Transcriptome Probe Set v2.0. Spatial alignment was conducted using high-resolution histology images and CytAssist images provided for each slide with default parameters. Gene expression matrices and spatial coordinate were generated at multiple spatial resolutions (2 μm, 8 μm, and 16 μm). Based on data quality, signal stability, and downstream interpretability, the 16 μm binned data were used for all subsequent analyses.

Quality control was performed at the spatial bin level using Seurat (v5.3.0). Bins were retained if they satisfied the following criteria: in_tissue = 1, nCount_Spatial >0, nFeature_Spatial >0, and mitochondrial gene content (percent.mt) < 15%. Mitochondrial gene percentages were calculated using genes annotated with the prefix “MT-“. Gene expression normalization was performed using the SCTransform method implemented in Seurat., applied to the Spatial assay without regressing out mitochondrial gene content. All genes were retained for downstream analyses by setting return.only.var.genes = FALSE. Bins with zero detected expression after initial SCTransform normalization (SCT = 0) were removed once, followed by re-normalization, and all subsequent analyses were conducted on the filtered dataset. To correct for slide-level batch effects, spatial transcriptomic data from two Visium HD slides were integrated using an SCT-based integration workflow. Integration features (n = 3000) were selected using SelectIntegrationFeatures, followed by PrepSCTIntegration. Integration anchors were identified with FindIntegrationAnchors using SCT normalization, and the datasets were integrated using IntegrateData.

Cluster-specific marker genes were identified using FindAllMarkers with the Wilcoxon rank-sum test on the SCT assay, with parameters set to only.pos = FALSE, min.pct = 0.01, and logfc.threshold = 0. Mitochondrial genes (prefix “MT-“) were excluded. Cell types were annotated based on well-established marker genes reported in previous human skin spatial transcriptomics and single-cell studies [52,53]. Epidermal compartments were defined as basal cells (TP63), spinous cells (KRT10 and KRTDAP), and granular cells (FLG), while fibroblasts were identified based on COL1A1 and TIMP1 expression. Marker expression patterns together with spatial localization were jointly considered for final annotation.

For selected senescence- and DNA damage-associated genes (CDKN1A, CRYAB, DST, EZR, FTL, HMOX1, KRT6A, S100A2, S100A16, and ZFP36), two spatial metrics were quantified: (i) the number of gene-positive bins and (ii) the density of gene-positive bins per unit tissue area (mm^2^). Gene positivity was defined as detectable expression in the SCT-normalized data with expression values greater than zero (SCT >0). Positive bins were counted among in-tissue bins (in_tissue = 1) for each gene within each condition. Tissue area was estimated from the Space Ranger in_tissue mask of the 16 μm binned outputs, assuming a bin area of 16 μm × 16 μm (0.000256 mm^2^ per bin). Spatial density was calculated as the number of positive bins divided by the total tissue area (mm^2^). Metrics were summarized at the condition level by pooling spatial bins across tissue sections within the same condition.

Differential expression analyses were performed using FindMarkers with the Wilcoxon rank-sum test on the SCT assay. Comparisons included negative control versus UV-damaged control (neg.ctrl vs. ctrl), and UV-damaged ICoN-treated versus UV-damaged control (ICoN vs ctrl). Parameters were set to min.pct = 0.01, logfc.threshold = 0, and only.pos = FALSE. All analyses were conducted using R (v4.5.1) in RStudio Server (v2025.05.0 Build 496). Key R packages included Seurat (v5.3.0), ggplot2 (v3.5.2), dplyr (v1.1.4), hdf5r (v1.3.12), and arrow (v20.0.0.2). Default parameters were used unless otherwise specified.

### 4.19 Lifespan and healthspan analysis with *C. Elegans*

*C. elegans* strains were cultured on Nematode Growth Medium (NGM) agar plates at 20 °C, with OP50 *Escherichia coli* provided as the primary food source. The Bristol N2 wild-type strain was acquired from the Caenorhabditis Genetics Center (CGC). To obtain synchronized nematodes, six unmated hermaphrodites were transferred to an NGM plate with an ample food supply. After four days, self-fertilized F1 progeny were collected, and synchronized hermaphrodite eggs were harvested and maintained on fresh NGM plates. OP50 bacteria were cultivated at 37 °C in LB medium for 12 h, and bacterial concentration was determined through colony counting and optical density (OD600) measurement.

Lifespan assays were performed at 20 °C using S-medium, which was supplemented with 3 mM CaCl_2_, 3 mM MgSO_4_, 50 μM EDTA, 25 μM FeSO_4_, 10 μM MnCl_2_, 1 μM CuSO_4_, 10 μM ZnSO_4_, and 10 mM KH_2_PO_4_ (pH 6.0), with OP50 bacteria as the nutritional source. ICoN was added to the medium at designated final concentrations. The assay followed standard liquid culture protocols and was conducted in three independent biological replicates. Synchronized *C. elegans* were prepared through timed egg laying (64 h) or direct egg synchronization, and approximately 150 young adults were transferred to fresh NGM plates. Living worms were subsequently moved to assay plates every other day, with fresh OP50 supplementation. Nematodes that left the assay plates or exhibited internal progeny hatching were excluded from analysis, while non-responsive worms, failing to react to gentle touch, were categorized as deceased. Lifespan data were processed using R software (version 4.1.0, “coin” package), with Kaplan-Meier survival curves and statistical significance assessed via the log-rank (Mantel-Cox) test.

For the oxidative stress resistance assay, all groups, including Neg. Ctrl (no treatment) and Ctrl (NAD^+^-free liposome), were exposed to 1 N hydrogen peroxide (Sigma-Aldrich, MO, USA) for 6 h on freshly prepared NGM plates. Twenty 10-day-old worms were used per group. The assay was conducted nine times, with fresh paraquat plates prepared immediately before each experiment. Each experimental condition was performed in triplicate, and survival rates were measured and statistically analyzed.

Age-related functional and metabolic parameters were assessed using a comprehensive healthspan analysis as previously described [54]. Briefly, worms were synchronized by standard bleaching and treated from the L4 larval stage (designated as day 0) with vehicle or the indicated compounds. 20 worms per group were analyzed for each assay. For lipofuscin assay, worms were collected at day 10 of adulthood, washed three times with M9 buffer, and transferred to black 96-well glass-bottom plates (Porvair Sciences). Whole-worm lipofuscin autofluorescence was measured using a multimode fluorescence plate reader (Synergy H1, BioTek). Signals were acquired using an excitation range of 390-410 nm and an emission range of 460-480 nm. Autofluorescence intensity was normalized to a stable intrinsic fluorescence signal obtained at excitation 280-300 nm and emission 320-340 nm to account for worm number and background variability. For locomotion analysis, worms at day 0 or day 10 adulthood were transferred to bacteria-free NGM plates and allowed to acclimate for 30 min at room temperature. Movement was recorded using a stereomicroscope system (Olympus SZ61) equipped with an eXcope T300 digital camera at 0.5-s intervals over a 20-s recording period. Five independent video segments were obtained per worm, and locomotor speed and body bending were calculated using TSView software (version 7.1). For triglyceride quantification, whole-worm lipid content was determined by measuring triglyceride levels using a colorimetric assay kit (Abcam), following the manufacturer's instructions with minor adaptations. Worm pellets were snap-frozen in liquid nitrogen, lysed in buffer containing 5% Triton X-100, and subjected to sonication. Samples were heated to 80 °C for 5 min with agitation to solubilize neutral lipids, cooled to room temperature, and centrifuged. Triglyceride content was normalized to total protein concentration measured by BCA assay (Pierce). Three independently prepared worm samples were analyzed per condition. For stress resistance assays, indicated as survival rate under heat stress, worms were exposed to 35 °C for 16 h, followed by assessment of viability.

### 4.20 Statistical analysis

All data are presented as mean ± standard deviation (s.d.). Statistical analyses were performed using R software (version 4.1.1) and GraphPad Prism (version 8.4.3). For comparisons between two groups, an unpaired two-tailed Student's t-test was used. For comparisons among three or more groups, one-way analysis of variance (ANOVA) followed by Tukey's multiple comparisons test was performed. The number of replicates (n) for each experiment is indicated in the corresponding figure legends, where the nature of the replicates (biological or technical) is specified for each experiment. Statistical significance was defined as ∗p < 0.05, ∗∗p < 0.01, ∗∗∗p < 0.001, and ∗∗∗∗p < 0.0001.

## Funding

This study was supported by 10.13039/501100017634LG Household and 10.13039/100018696Health Care R&D Center, GIST Future-Leading Specialized Research Project (GKH0900 to D.R.), and a grant from the 10.13039/501100003725National Research Foundation of Korea (10.13039/501100003725NRF), funded by the 10.13039/501100014188Ministry of Science and ICT (2022K2A9A1A06091879, 2023R1A2C3006220 to D.R.). Furthermore, we acknowledge the equipment and technical support provided by the GIST Advanced Institute of Instrumental Analysis (GAIA).

## CRediT authorship contribution statement

**Seongsu Kang:** Data curation, Writing – original draft. **Shibo Wei:** Data curation, Writing – original draft. **Bon Il Koo:** Data curation, Writing – original draft. **Yunju Jo:** Methodology, Software. **Junhyeon Park:** Software. **Yingqi Xue:** Data curation, Formal analysis. **Sanghyun Ye:** Investigation, Methodology. **Jiwon Park:** Validation. **Byung Woo Hwang:** Visualization. **Jin Hyun Kim:** Data curation. **Euitaek Jeong:** Investigation. **Juewon Kim:** Data curation, Supervision. **Nea-Gyu Kang:** Conceptualization, Project administration, Supervision. **Seung-Hyun Jun:** Conceptualization, Project administration, Writing – review & editing. **Dongryeol Ryu:** Conceptualization, Project administration, Writing – review & editing.

## Declaration of competing interest

The authors declare the following financial interests/personal relationships which may be considered as potential competing interests:

S.K., B.I.K., S.Y., J.P., B.W.H., J.H.K., E.J., N.G.K., and S.H.J. are employees of LG Household & Health Care R&D Center. If there are other authors, they declare that they have no known competing financial interests or personal relationships that could have appeared to influence the work reported in this paper.

## Data Availability

Data will be made available on request.
