# Supplementary material for: Ion-coupled transfersome complexes for enhanced transdermal NAD+ repletion and mitigation of cellular senescence signatures
Source: Mater Today Bio. 2026 May 6;38:103218. doi: 10.1016/j.mtbio.2026.103218 (PMC13188140; doi:10.1016/j.mtbio.2026.103218)
Supplement: Multimedia component 1 [file mmc1.docx]

**Supplementary Materials** for

Ion-coupled Transfersome Complexes for Enhanced Transdermal NAD^+^ Repletion and Mitigation of Cellular Senescence Signatures

Seongsu Kang †, Shibo Wei †, Bon Il Koo †, Yunju Jo, Junhyeon Park, Yingqi Xue, Sanghyun Ye, Jiwon Park, Byung Woo Hwang, Jin Hyun Kim, Euitaek Jeong, Juewon Kim, Nea-Gyu Kang *, Seung-Hyun Jun *, and Dongryeol Ryu *

*** Correspondence to** [dryu@gist.ac.kr](mailto:dryu@gist.ac.kr), [junsh@lghnh.com](mailto:junsh@lghnh.com), [ngkang@lghnh.com](mailto:ngkang@lghnh.com)

**This PDF file includes:**

Figures S1 to S13

Tables S1 to S2


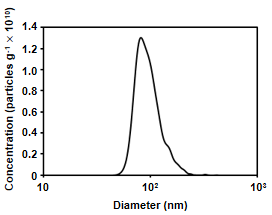


**Fig. S1. Size distribution of ICoN from nanoparticle tracking analysis (NTA).**


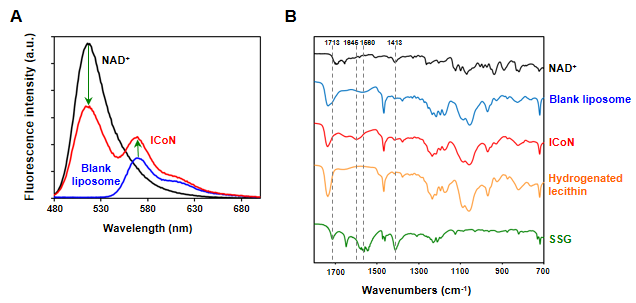


**Fig. S2.** **Experimental evidence supporting NAD^+^-liposome interactions in ICoN.** (A) Fluorescence emission spectra of NAD^+^ (black), blank liposomes (blue), and ICoN (red) (λ_ex = 450 nm), demonstrating FRET-based proximity between NAD^+^ and the liposomal surface. (B) FTIR spectra of NAD^+^ (black), blank liposomes (blue), ICoN (red), hydrogenated lecithin (yellow), and sodium stearoyl glutamate (SSG) (green), showing changes in carboxyl group–related vibrational regions upon incorporation of NAD^+^.


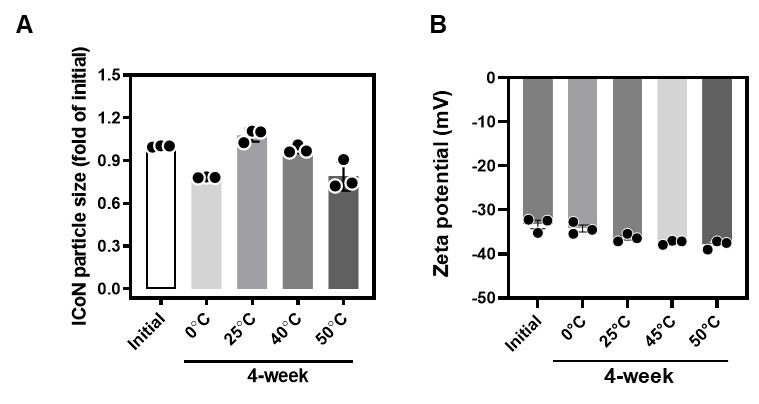


**Fig. S3. Long-term Physicochemical Stability of ICoN Under Varied Storage Conditions.** Hydrodynamic particle size of ICoN measured over a 4-week period under different temperature conditions (n=3, biological replicates). Particle size (A) and zeta potential (B) of ICoN after long-term storage. Corresponding PDI values are provided to reflect changes in size distribution under each condition (initial: 0.164 ± 0.023; 0°C: 0.146 ± 0.052; 25°C: 0.198 ± 0.029; 40°C: 0.200 ± 0.013; 50°C: 0.214 ± 0.017).


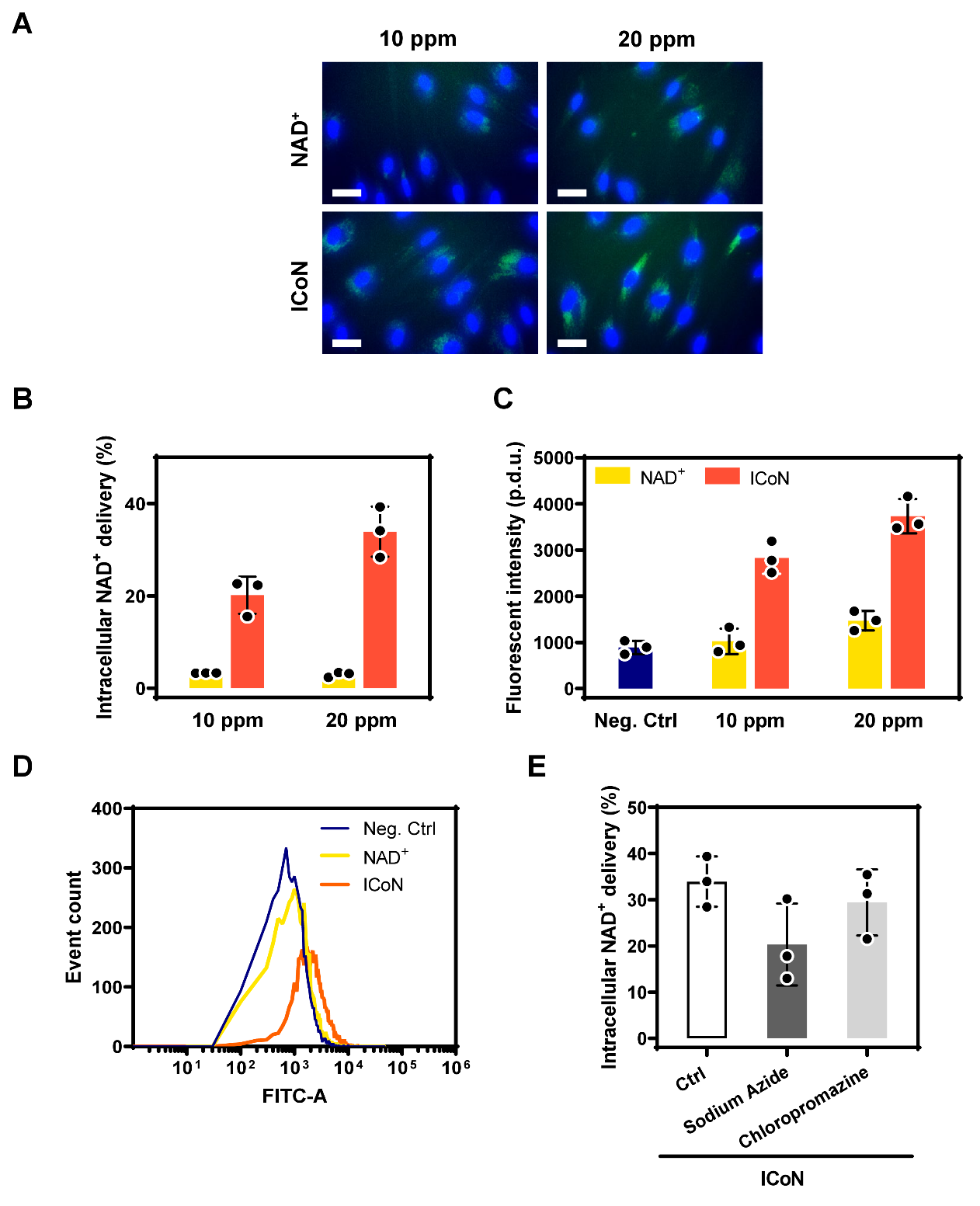


**Fig. S4. ICoN Enhances Intracellular Delivery of NAD^+^ Independently of Classical Endocytic Pathways.** (**A**) Representative fluorescence microscopy images of cells treated with FITC-labeled NAD^+^ delivered either as free NAD^+^ or via ICoN at 10 or 20 ppm. Nuclei were counterstained with DAPI. Scale bar = 20 μm. (**B-C**) Quantification of intracellular NAD^+^ delivery efficiency (quantified by enzyme-linked immunosorbent assay, B) and FITC fluorescence intensity (C) (n=3, biological replicates). (**D**) Flow cytometric analysis of FITC fluorescence in cells treated with free NAD^+^ or ICoN. (**E**) Effects of pharmacological inhibition of endocytic pathways on ICoN-mediated NAD^+^ delivery (n=3, biological replicates).


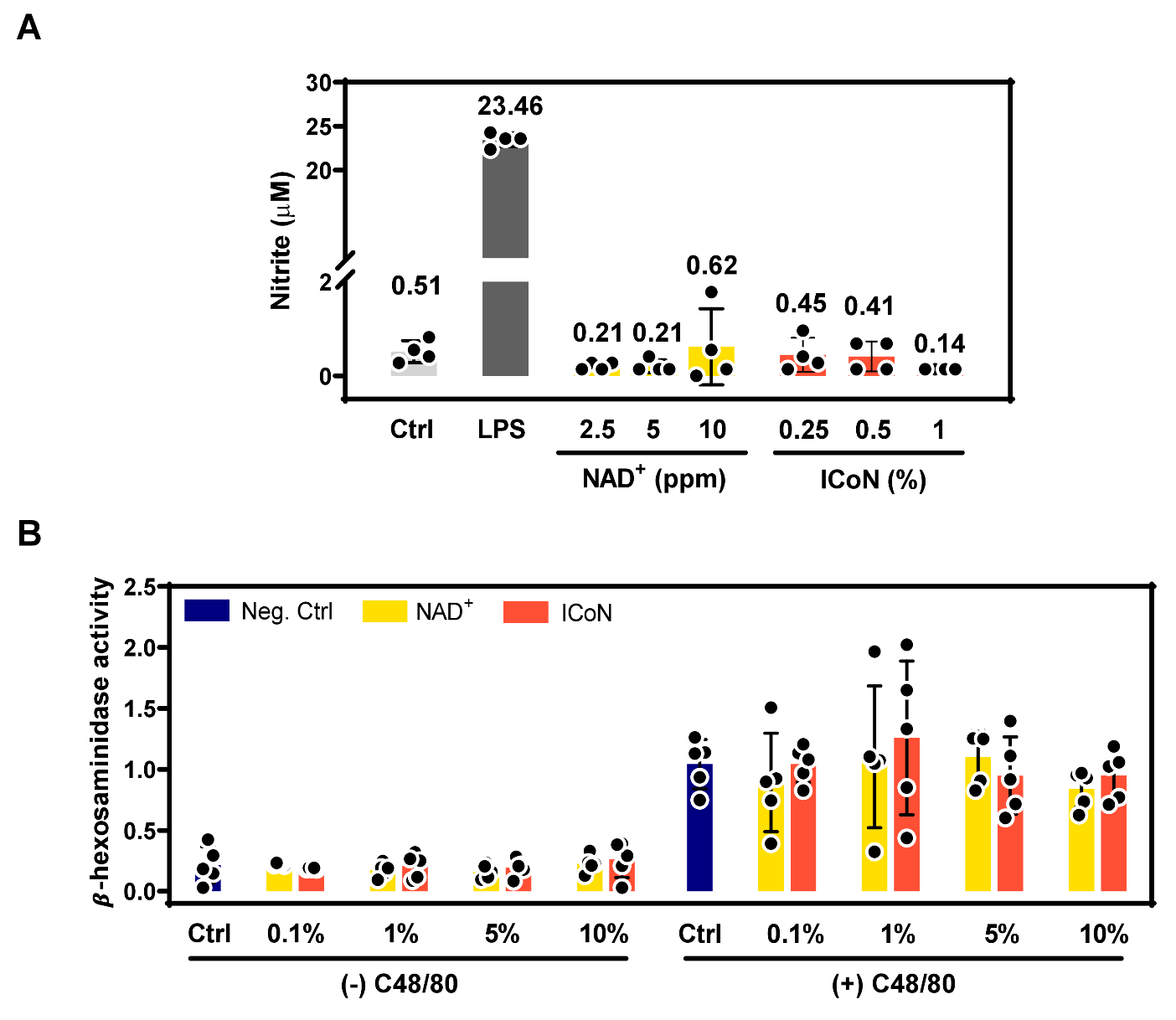


**Fig. S5. Evaluation of Immunological Compatibility of ICoN During NAD^+^ Delivery.** (**A**) Nitrite release from RAW264.7 macrophages following treatment with free NAD^+^ or ICoN at different concentrations (n=4, biological replicates). (**B**) β-hexosaminidase activity from RBL-2H3 mast cells following exposure to free NAD^+^ or ICoN, with or without C48/80 treatment, assessed as an indicator of mast cell degranulation (n=5, biological replicates).


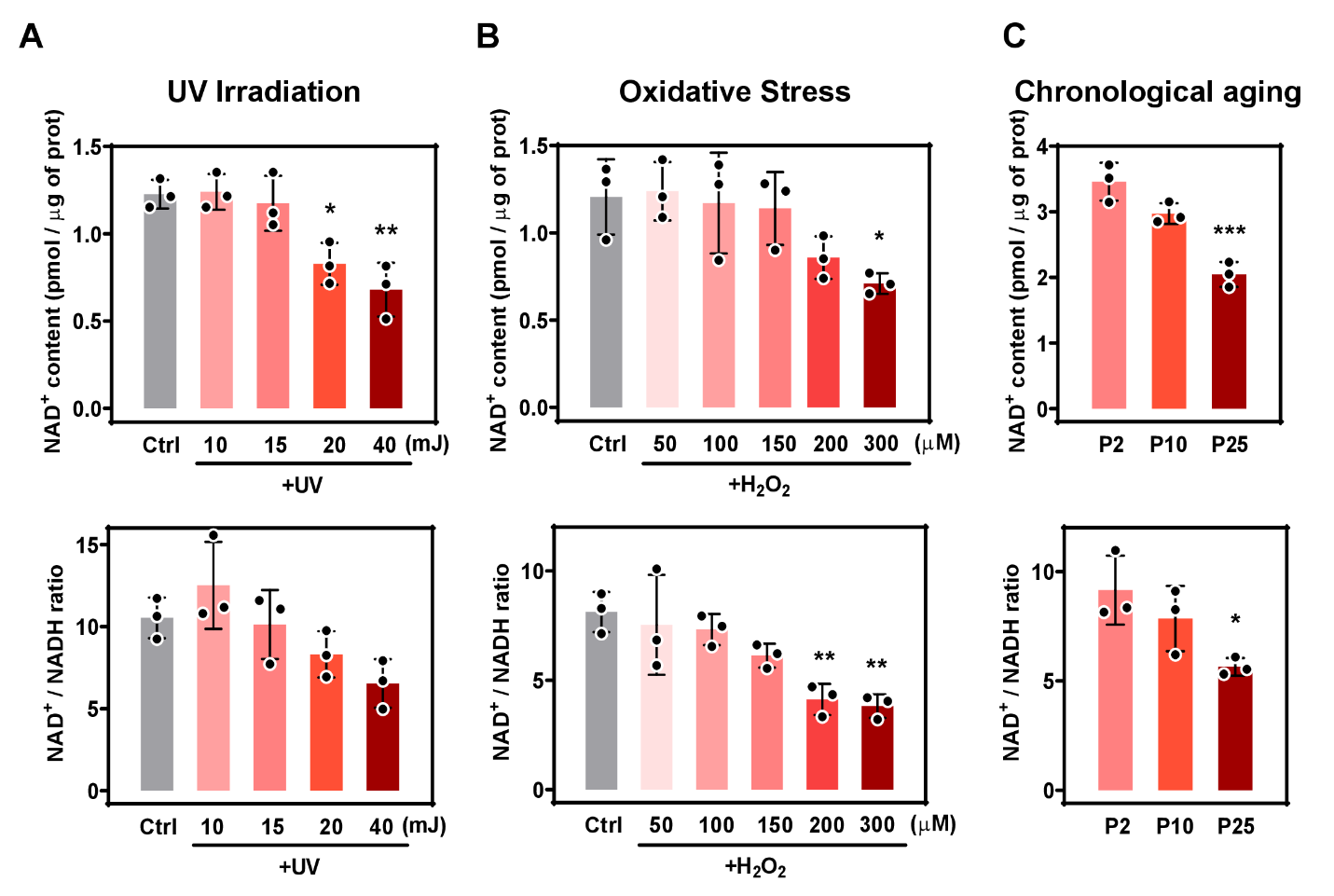


**Fig. S6.** **Alterations in intracellular NAD^+^ levels in human skin fibroblasts under various aging conditions.** Intracellular NAD^+^ concentrations were quantified in skin fibroblasts (Hs68) subjected to different aging models, including UV irradiation (**A**), oxidative stress (**B**), and replicative senescence (**C**). “P” refers to passage of cell cultures. Cellular NAD^+^ levels were measured 24 hours after treatment with UV and H_2_O_2_. Data are presented as mean ± SEM (n = 3, biological replicates).


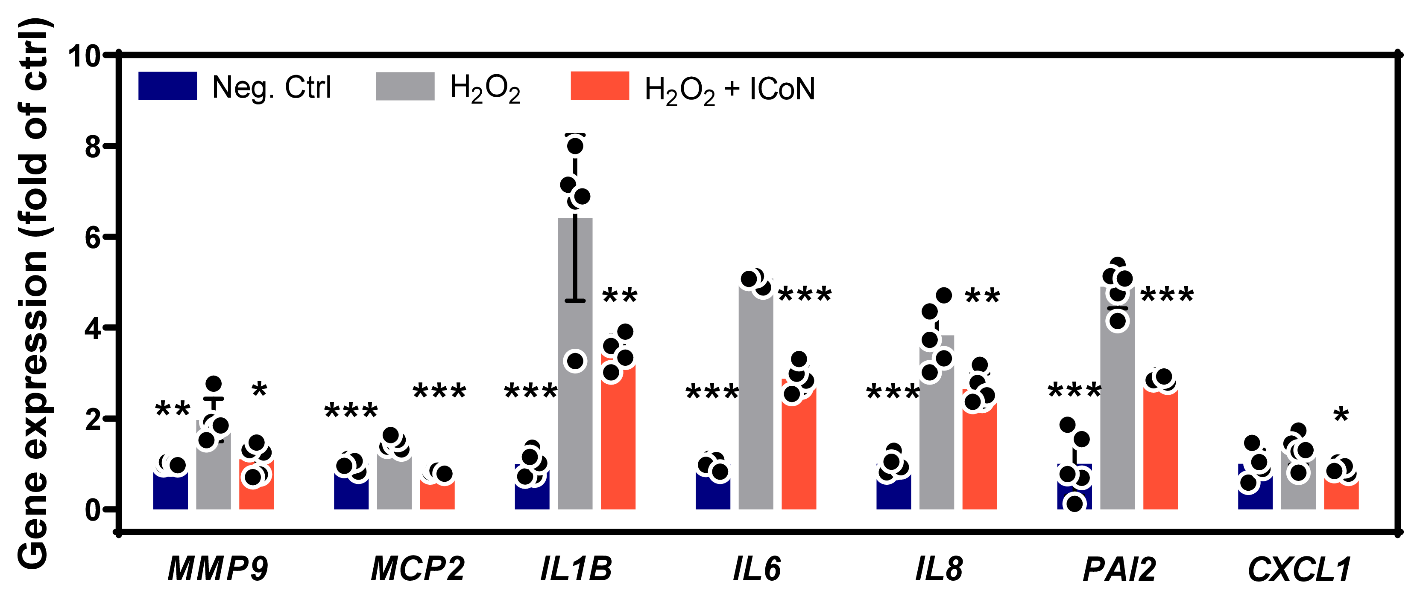


**Fig. S7. ICoN Suppresses the Expression of Senescence-Associated Secretory Phenotype Markers Induced by Oxidative Stress.** Relative mRNA expression levels of *MMP9, MCP2, IL1B, IL6, IL8, PAI2,* and *CXCL1* were quantified using RT-qPCR in cells treated with H_2_O_2_-induced oxidative stress and supplemented with ICoN (n = 5, biological replicates).


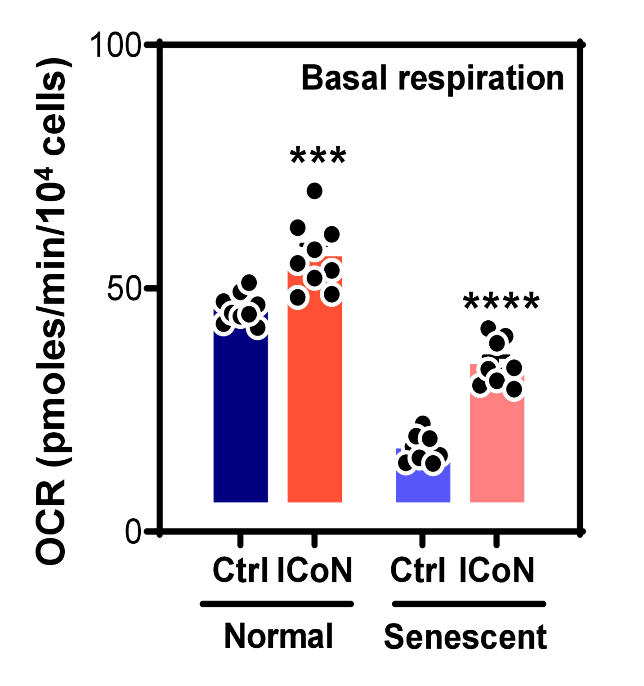


**Fig. S8. Quantification of Mitochondrial Basal Respiration.** Basal mitochondrial oxygen consumption rate was measured in normal and senescent cells with or without ICoN treatment (n=9, biological replicates).


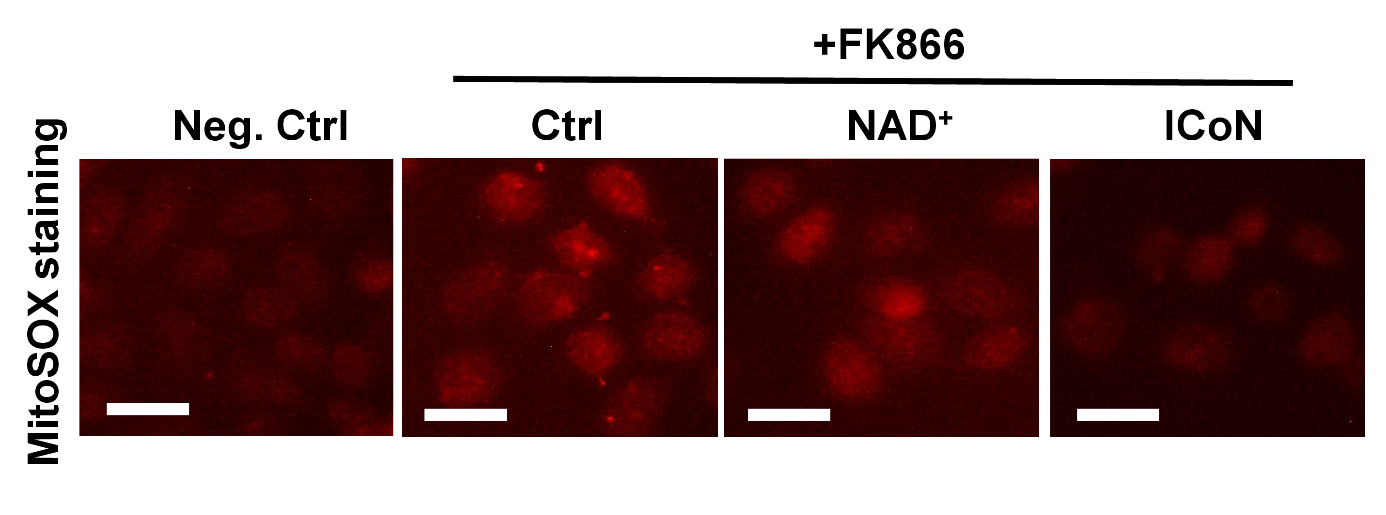


**Fig. S9. Fluorescent Images of MitoSOX Staining.**


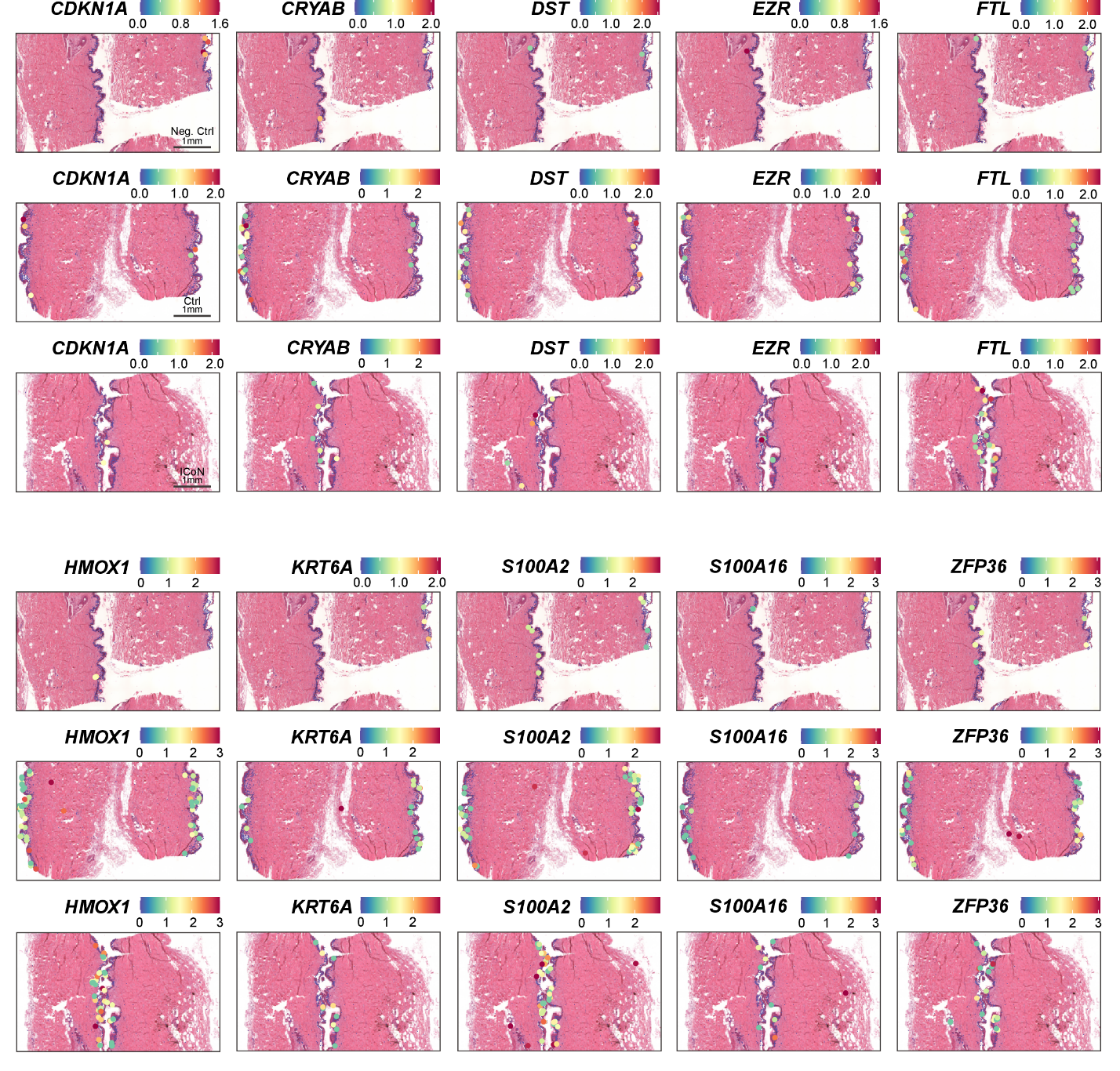


**Fig. S10. Spatial Feature Plots of Senescence- and DNA Damage-Associated Genes in *Ex Vivo* Human Skin Tissue.** Representative spatial feature plots showing SCT-normalized expression of *CDKN1A, CRYAB, DST, EZR, FTL, HMOX1, KRT6A, S100A2, S100A16*, and *ZFP36* across *ex vivo* human skin explants under three conditions: negative control (Neg. ctrl, upper), UV-exposed control (Ctrl, middle), and UV-exposed with ICoN treatment (ICoN, bottom). Gene expression is overlaid on H&E-stained histological images using 16 μm spatial bins, with color intensity indicating normalized expression levels (SCT assay). Scale bar = 1 mm.


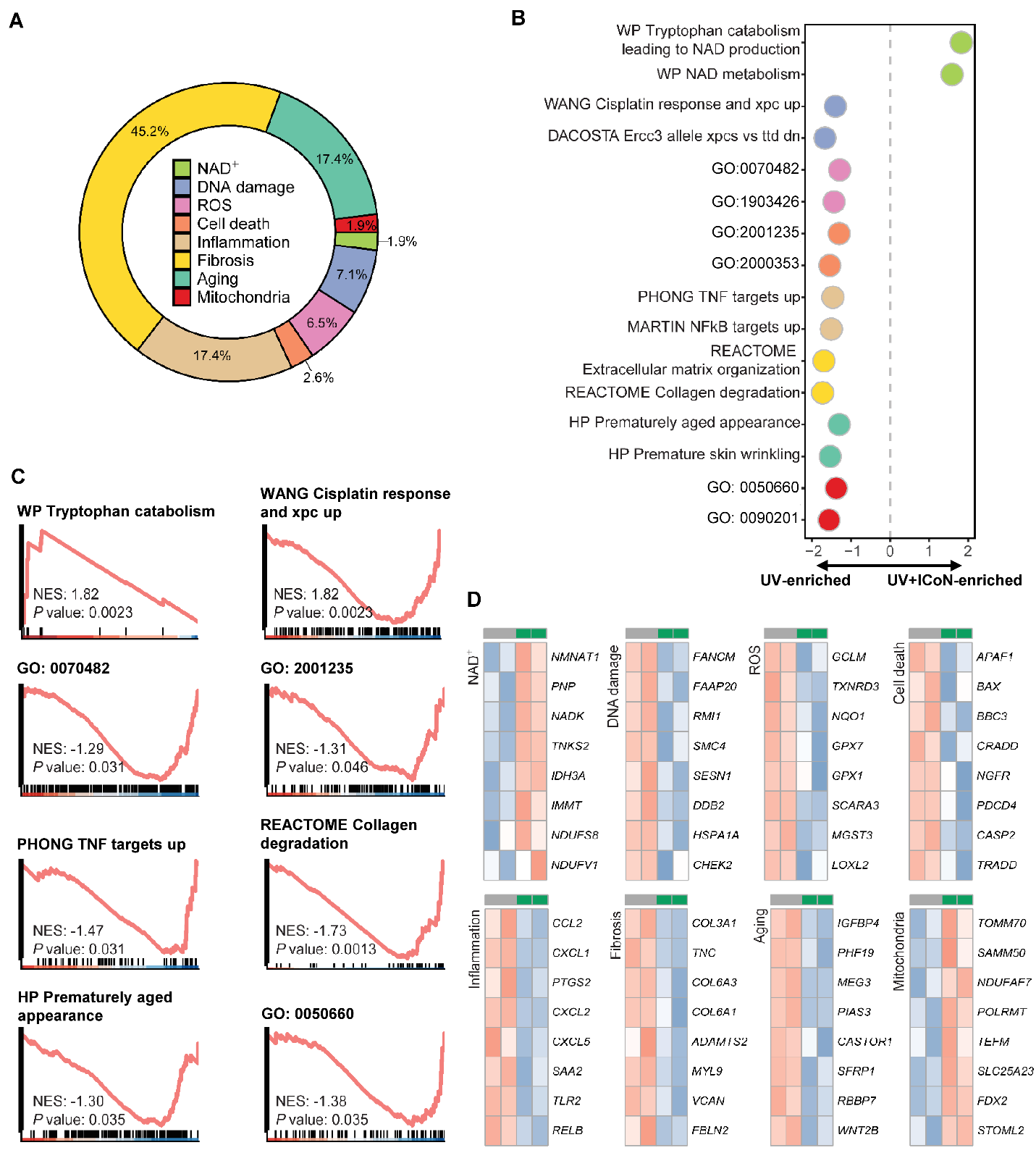


**Fig. S11. ICoN Reverses Photoaging-Associated Transcriptional Dysregulation in *Ex Vivo* Human Skin Explants.** (**A**) Pie chart summarizing significantly enriched gene sets identified by GSEA comparing the UV and UV + ICoN groups. The gene sets were categorized into eight functional themes. The slices are arranged clockwise beginning from the NAD^+^ category. (**B**) Bubble plot showing enriched pathways from the UV versus UV + ICoN GSEA. Dot colors indicate the functional category of each gene set: NAD^+^ (light green), DNA damage (pastel blue), ROS (pink), cell death (orange), inflammation (beige), fibrosis (yellow), aging (mint), and mitochondria (red). The x-axis represents the normalized enrichment score (NES), where positive values indicate enrichment in the UV + ICoN group and negative values indicate enrichment in the UV group. (**C**) Gene set enrichment plots for the selected pathways. The normalized enrichment score (NES) and P-value indicate the magnitude and significance of pathway activation. For visualization clarity, gene sets with long names were truncated. WP Tryptophan catabolism leading to NAD production, WANG Cisplatin response and XPC up, GOBP Response to oxygen levels, GOBP Positive regulation of apoptotic signaling pathway, PHONG TNF targets up, REACTOME Collagen degradation, HP Prematurely aged appearance, GOMF Flavin adenine dinucleotide binding. (**D**) Heatmaps of representative genes selected from each gene set. Gene expression levels (log₂[tpm+1]) were standardized as Z-scores to visualize the differences between the UV group (gray) and the UV + ICoN group (green).


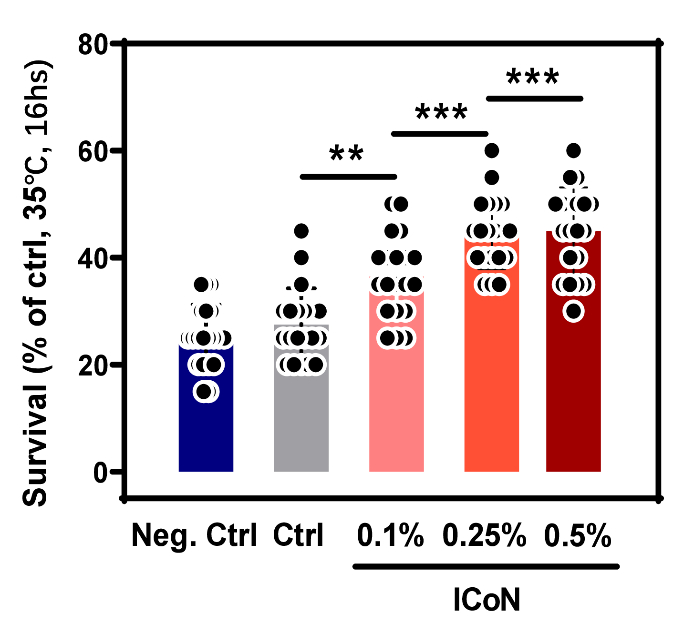


**Fig. S12. ICoN Enhances Heat Stress Resistance in *C. elegans*.** Survival rate of *C. elegans* was quantified following exposure to acute heat stress at 35 °C (n=20, biological replicates).


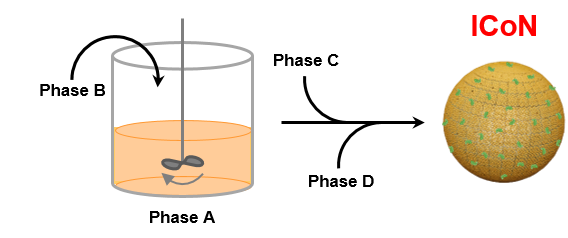


**Fig. S13. Preparation of Ion-Coupled NAD^+^ Transferosome Complexes (ICoN).**

**Table S1. Gene sets enriched in UV-irradiated + ICoN vs. UV-irradiated Group identified by GSEA.**

|  | GO Number | GO Term Name |
| --- | --- | --- |
| ROS | GO:0070482 | GOBP Response to oxygen levels |
|  | GO:1903426 | GOBP Regulation of reactive oxygen species biosynthetic process |
| Cell death | GO:2001235 | GOBP Positive regulation of apoptotic signaling pathway |
|  | GO:2000353 | GOBP Positive regulation of endothelial cell apoptotic process |
| Mitochondria | GO:0050660 | GOMF Flavin adenine dinucleotide binding |
|  | GO:0090201 | GOBP Negative regulation of release of cytochrome c from mitochondria |

**Table S2. Compositions of ICoN.**

| **Phase** | **Materials** | **Contents (%)** |
| --- | --- | --- |
| A | Dipropylene glycol | 1.68 |
|  | Hydrogenated lecithin | 0.05 |
|  | Cholesterol | 0.01 |
|  | Sorbitan oleate | 0.01 |
|  | Sodium stearoyl glutamate | 0.01 |
| B | Deionized water | 3.33 |
| C | Deionized water | 44.93 |
| D | NAD^+^ | Adjust according to NAD^+^ contents |
|  | Deionized water |  |
|  | 1,2-Hexanediol | 2.50 |
|  | Total | 100 |
